# Behavioural correlates of combinatorial versus temporal features of odour codes

**DOI:** 10.1038/ncomms7953

**Published:** 2015-04-27

**Authors:** Debajit Saha, Chao Li, Steven Peterson, William Padovano, Nalin Katta, Baranidharan Raman

**Affiliations:** 1 Department of Biomedical Engineering, Washington University in St Louis, St Louis, Missouri 63130, USA

## Abstract

Most sensory stimuli evoke spiking responses that are distributed across neurons and are temporally structured. Whether the temporal structure of ensemble activity is modulated to facilitate different neural computations is not known. Here, we investigated this issue in the insect olfactory system. We found that an odourant can generate synchronous or asynchronous spiking activity across a neural ensemble in the antennal lobe circuit depending on its relative novelty with respect to a preceding stimulus. Regardless of variations in temporal spiking patterns, the activated combinations of neurons robustly represented stimulus identity. Consistent with this interpretation, locusts reliably recognized both solitary and sequential introductions of trained odourants in a quantitative behavioural assay. However, predictable behavioural responses across locusts were observed only to novel stimuli that evoked synchronized spiking patterns across neural ensembles. Hence, our results indicate that the combinatorial ensemble response encodes for stimulus identity, whereas the temporal structure of the ensemble response selectively emphasizes novel stimuli.

## Abstract (web summary)

In the olfactory system, odourants typically evoke spiking responses in neurons that are both spatially and temporally structured. Here, the authors demonstrate that odour identity is encoded purely by the combinations of neurons activated and is insensitive to changes in temporal structure.

## Graphical abstract

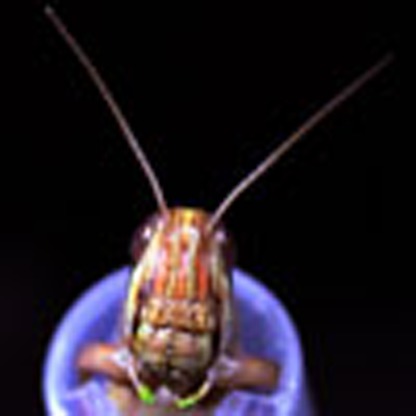

Time and space are fundamental neural coding dimensions. Sensory cues, even stationary ones, often activate an ensemble of neurons with a precise temporal structure. Determining what features of a stimulus are encoded by the active set of neurons (‘combinatorial code') and what aspects are represented in their temporal structure (‘temporal code') is a fundamental problem in systems neuroscience. Alternatively, these two dimensions may not independently encode information. In this case, the joint spatiotemporal patterns of spiking activity could provide a large coding space for representing stimuli^1^,^2^. Identifying the right coding scheme employed by a sensory system is essential for determining the rules that govern how stimulus-evoked neural activity is translated to a behavioural response.

In the insect olfactory system, olfactory sensory neurons in the antenna transduce chemical cues into electrical signals and transmit them to the antennal lobe neural circuits (analogous to the olfactory bulb in vertebrates) for further processing^1^,^2^,^3^,^4^. Previous studies have shown that odourants activate temporally structured principal neuron responses in the antennal lobe (and in the olfactory bulb) that vary with and therefore have the capacity to encode for stimulus identity and intensity^3^,^4^,^5^. However, these response patterns are disrupted by hysteresis arising from stimulus dynamics^6^,^7^ and recent history^8^. Furthermore, to date there is no behavioural evidence to suggest that insects use the temporal structure of neural responses for odour recognition.

On the other hand, a purely combinatorial code offers greater encoding capacity than a labelled-line scheme where a set of neurons exclusively represents a stimulus. However, adapting the sensory system to ignore a redundant stimulus becomes difficult, as suppressing responses to one odourant will also alter the neural activity evoked by a number of other stimuli. How then can the system preserve the identity of a cross-adapted odourant?

Here we sought to investigate these issues using the locust olfactory system. We show that although combinatorial and temporal codes by themselves have potential deficiencies, together they can allow robust encoding of stimulus identity and facilitate emphasizing or deemphasizing certain stimuli based on their novelty or lack thereof. Furthermore, we reveal how information contained in the combinatorial and temporal features of stimulus-evoked activities gets translated to behaviour.

## Results

### Stimulus history can disrupt temporal response patterns

We began by analysing the responses of projection neurons (PNs) in the antennal lobe to lengthy but solitary presentations of different odourants. Both simple (monomolecular) and complex cues were used. In general, we found that the onset of stimulus-evoked activity was rapid with 60–90% of responsive neurons reaching above baseline levels within 600 ms of odourant onset (Supplementary Table 1; latency is defined as the time to reach 20% of the peak response levels; see Methods). The median response latency was in 300–400 ms range for all odourants used except citral that elicited a comparatively slower response (550 ms median latency).

**Figure 1 f1:**
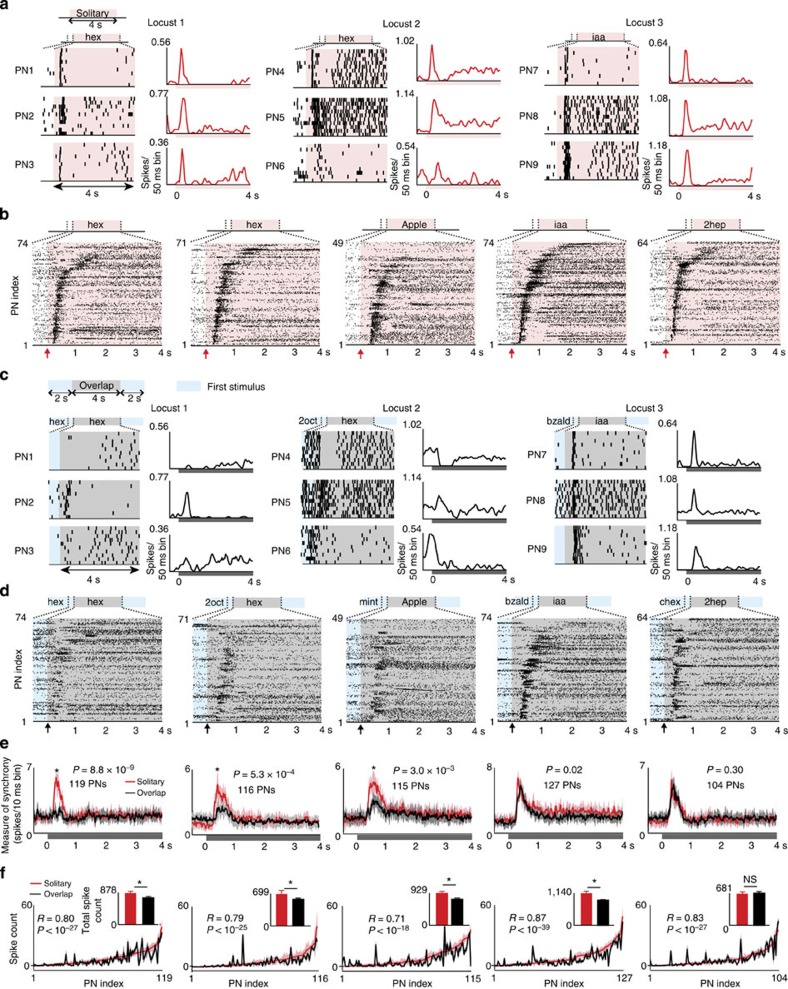
Stimulus history alters the temporal structure of ensemble responses in the antennal lobe. (**a**) Projection neuron (PN) spiking responses evoked by solitary introductions of different odourants are shown for ten consecutive trials. Each row reveals spiking patterns observed during an initial 500 ms prestimulus period (unshaded portions), followed by a four seconds of odour-evoked activity (shaded portions). The mean spike counts as a function of time (peristimulus time histogram (PSTH); 50 ms time bins) are shown in the right panel. Note that the three PNs shown in a column were simultaneously recorded from a single locust. (**b**) Spiking activities of PNs (pooled across experiments; 10 trials each) with significant responses to the following odourants: hexanol (hex), apple, isoamyl acetate (iaa) and 2-heptanone (2hep) are shown. PNs were sorted based on their average latency to peak response. Arrows along *x*-axis indicate the time of odour onset. Note that the two different PN ensemble responses shown for hexanol were probed with different odourant sequences. (**c**) The trial-by-trial spike rasters and mean firing rates of the same set of PNs to the same set of odourants as in **a** are shown. However, the odourants are now presented in an overlapping sequence with another preceding stimulus with a 2-s delay. The 4-s period of stimulus overlaps are shaded in grey, whereas a blue shade is used to represent a 500-ms time period when the first stimulus in the sequence was present alone. (**d**) Responses of PNs to the following two-odour overlapping sequences: hexanol–2 s latency–hexanol (hex(hex)), 2octanol–2 s latency–hexanol (hex(2oct)), mint–2 s latency–apple (apple(mint)), benzaldehyde–2 s latency–isoamyl acetate (iaa(bzald)), and cyclohexanone–2 s latency–2heptanone(2hep(chex)). Same PN order as in **b**. (**e**) Red traces represent spike counts across all PNs following solitary introductions of an odourant, and the black traces show the spike counts across the same set of PNs following introductions of the same odourant but presented in an overlapping sequence (mean±s.d.; *n*=10 trials, bin size=10 ms). Asterisks indicate significant reductions in peak spike counts (*P<0.01, paired *t*-tests, *n*=10 trials). (**f**) Spike counts summed over the entire duration of odour presentation (4 s) are shown for all PNs during solitary (in red) and overlapping (in black) stimulus conditions (mean±s.d.; *n*=10 trials). Correlation coefficients (*R*) were calculated between PN response profiles shown in black and red. *P* indicates the significance level of the observed correlation. Insets show the total spike-count across all PNs during solitary and overlapping presentation of the same odour (mean±s.d.). Asterisks indicate significant change in total spike counts (*P<0.01, NS: *P*>0.01, paired *t*-tests, *n*=10 trials).

We found that lengthy odour exposures generated temporally patterned spike trains in individual PNs (Fig. 1a,b), and that different neurons had different patterns of stimulus-evoked responses. For example, the spike rasters for PN4–PN6 for Locust 2 (Fig. 1a, middle column) exhibit markedly different activity patterns in response to the same odourant (hexanol). However, as an ensemble, the PNs tended to reach their peak response values in a coherent manner (Supplementary Fig. 1a,b). This was true whether the neurons were recorded simultaneously from a single locust (Fig. 1a), or pooled across multiple animals (Fig. 1b). As a consequence, during this period of high stimulus-evoked activity, coincident spikes across PNs were observed for a wide variety of chemical cues tested. Qualitatively, the number of neurons that contributed to synchronous firings and the time periods when such activities were observed for each odourant can be identified by vertical alignment of tick marks in the raster plots showing spiking activity across neurons (Fig. 1b and Supplementary Fig. 1c).

In natural conditions, odourants are seldom encountered in isolation and are typically preceded and succeeded by other chemosensory cues. Could the observed neural response coherence be disrupted when odourants are not encountered in isolation but presented following another stimulus with some overlap? To examine this, we delivered the same set of stimuli in an overlapping sequence preceded by another pulse of the same or a different odourant. We delayed introduction of the second pulse until the response to the first pulse had reached a low, but persistent state (or ‘steady-state' activity^9^,^10^,^11^). Note that the first stimulus was not terminated before or during the second stimulus exposure. This was done to prevent offset responses due to termination of the first stimulus from combining with responses evoked by the succeeding stimulus in the sequence. This stimulation protocol allowed us to compare the ensemble spiking responses following solitary introduction of a stimulus with those elicited by the same stimulus when delivered in an overlapping sequence.

We found that temporal structure of the spike trains generated by an odourant could change depending on whether the stimulus was presented in isolation or introduced following another odourant (Fig. 1a versus Fig. 1c; note, we refer to the activity following the introduction of the second pulse as second-odourant (first-odourant)). Interestingly, for some odour sequences (for example, iaa versus iaa(bzald)), the temporal response patterns were robustly maintained.

At the neural ensemble level, as expected, the spiking responses to the onset of the first odourant were synchronized. However, in most cases, the temporal structures of neural activities following the introduction of the second stimulus in the sequence were less coherent across neurons compared with the responses elicited during solitary introductions (Fig. 1c,d and Supplementary Fig. 2a, b). We found that different neurons reached their peaks at different time points (Supplementary Fig. 3). Hence, the second odourant in the sequence was less likely to elicit coincident spikes across PNs (Fig. 1c,d).

To quantify these results, we counted the number of spikes observed across the neural ensemble in 10 ms non-overlapping time bins (red traces in Fig. 1e and Supplementary Fig. 2c). Note that coincident spikes across neurons result in the larger spike counts in a given time bin and therefore can be used as a measure of neural response synchrony. These results confirm that solitary presentations of any odourant generated synchronous spikes across an ensemble of PNs in the antennal lobe. Compared with responses evoked during solitary presentations, the degree of spike synchrony for most odourants was significantly reduced when they were introduced following another stimulus (black traces in Fig. 1e and Supplementary Fig. 2c; paired *t*-test, * indicates *P*<0.01, *n*=10 trials). Surprisingly, for three out of the nine odour sequences presented in this manner, the ensemble response to the second pulse was as synchronous as the responses evoked by their solitary presentations (refer to iaa(bzald) and 2hep(chex) cases in Fig. 1; also refer to hex(hxa) case in Supplementary Fig. 2). These results were independent of the size of the time-bin used for analysis (Supplementary Fig. 4) and were qualitatively similar to those obtained using simultaneously recorded neurons from a single locust (Supplementary Fig. 5). Taken together, these results indicate that the same set of stimuli can generate neural activities with different temporal structures in the antennal lobe depending on whether they were introduced solitarily or in an overlapping sequence.

### Combinatorial odour code robustly encodes odour identity

Next, we examined whether the activated set of neurons was altered between solitary versus overlapping presentations of the same odourant. We computed the spike counts of each individual PN during the entire odour duration, that is, following solitary introduction of the odourant or following the introduction of the same odourant as the second stimulus in the sequence. We then compared the ensemble activation profiles across the two presentation conditions (Fig. 1f; red (solitary) versus black (overlap) curves). Although there were some variations, the overall PN ensemble activation profile during solitary and overlapping introductions was surprisingly well conserved for most odourants (Fig. 1f and Supplementary Fig. 2d). Furthermore, the correlation between ensemble activation profiles across different presentation conditions was comparable to those observed across different trials of the same stimulus (Supplementary Fig. 2e; two-way analysis of variance (ANOVA) with Bonferroni correction for multiple comparisons, *P*=0.12). Correlations across different trials and across different conditions were significantly higher than those observed between response profiles generated by different odourants (Supplementary Fig. 2e; two-way ANOVA, *P*(inter-trial versus inter-odour)*=*1.53 × 10^−5^*, P*(inter-condition versus inter-odour)*=*7.25 × 10^−4^).

Hence, our results indicate that with respect to variations in how a stimulus is encountered the combinatorial features of ensemble activity are more robust than temporal response patterns.

### Response magnitude versus neural synchrony

It is possible that the reduction in spike synchrony across neurons may simply arise due to diminished spike counts observed during sequential introduction of some odourants. To test this hypothesis, we compared the spike counts elicited by an odourant during solitary versus overlapping presentation conditions. At the individual PN level, we found that the total spike counts could significantly increase/decrease or remain consistent across presentation conditions (Supplementary Fig. 6a; paired t-test, at 0.05 confidence level). The distributions of number of PNs with increase/decrease/no change across presentation conditions were comparable across different odour pairs used in the study (Supplementary Fig. 6b; two-way ANOVA, *P*=1.0).

**Figure 2 f2:**
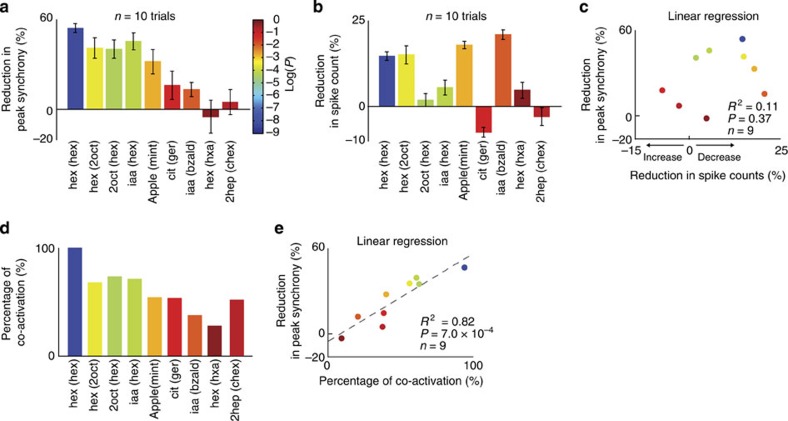
Rules for cross-talk between odour-evoked responses in the antennal lobe. (**a**) Percentage reduction in the peak spike synchrony between solitary versus overlapping introductions of the same odourant is shown (mean±s.e.m.; *n*=10 trials). The bars are shaded based on the significance of peak neural synchrony reduction (logarithms of their *P* values). (**b**) Percentage reduction in total spikes across neurons is shown for solitary versus overlapping introduction of the same odourant (area under black curve/area under red curve shown in Fig. 1f). Same colour convention as in **a**. (**c**) Regression analysis between spike-count reduction (*x*-axis; **b**) and reduction in peak synchrony (*y*-axis; **a**) did not reveal any significant relationship between variables (*R*^2^=0.11, *P*=0.37). (**d**) Percentage of neurons activated by solitary presentations of both odourants is shown (see Methods). Same colour convention as in **a**. (**e**) Regression analysis between response overlap as quantified by the percentage of co-activation (*x*-axis; **d**) and reduction in neural response synchrony (*y*-axis; **a**) revealed a significant linear relationship between the two variables (*R*^2^=0.82, *P*=7 × 10^−4^).

At the ensemble level, we again found that the total spike counts across neurons can also significantly increase/decrease or remain consistent across presentation conditions (Fig. 1f insets; area under the red/black curves; also refer Supplementary Fig. 2d insets; paired *t*-tests, *P<0.01). Notably, even when the overall spike synchrony did not diminish (for example, iaa versus iaa(bzald)), we found that the total spike counts across neurons can decrease significantly during overlapping presentations of the stimulus (Fig. 2b). Alternately, we found cases where the overall spike synchrony reduced significantly (for example, 2oct versus 2oct(hex)), but the total spike counts across neurons remained consistent across conditions (Fig. 2b and Supplementary Fig. 2d).

We found that there was no significant correlation between reduction in spike synchrony and reduction in spike counts (Fig. 2a–c; linear regression analysis, *R*^*2*^=0.11, *P=*0.37). Hence, we conclude that the observed reduction in the spike synchrony across neurons (Fig. 1e and Supplementary Fig. 2c) cannot be explained based on changes in the response magnitude during solitary versus overlapping introductions of the same stimulus.

### Cross-talk between stimulus-evoked responses

Are there rules that govern when the response to a preceding stimulus diminishes the coherence in ensemble activity evoked by a succeeding stimulus? We hypothesized that this cross-talk between neural activities evoked by the two stimuli could be a result of their response overlap. Therefore, we first computed the percentage of PNs that were activated by both odourants presented in a sequence (see Methods). As odourants evoked a combinatorial response in this network, in general, we found that any two odourants had some amount of overlap. However, for those odour pairs where disruption in synchrony was observed, we found that the ensemble response overlap was considerably greater (Fig. 2a,d). A significant linear relationship was observed between PN response overlap and reduction in neural synchrony (Fig. 2e; linear regression analysis, *R*^*2*^=0.82, *P*=7 × 10^−4^).

Notably, this cross-talk was not determined by chemical similarity (same versus different functional groups), behavioural similarity (geraniol is an attractant and citral is a repellant^12^) or odour delivery sequence (hex(2oct) versus 2oct(hex)), but was purely determined by the neural response overlaps (an inverse measure of the novelty of the stimulus). Hence, these results reveal that when encountering a series of stimuli, only those odourants that were less redundant, or more novel, evoked synchronous activity in the antennal lobe.

### Odour identity can be robustly maintained across conditions

**Figure 3:**
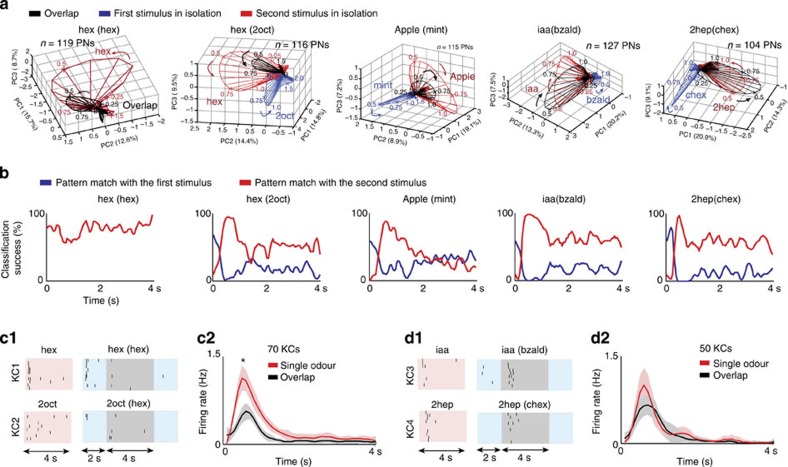
Desynchronization reduces response intensity without altering stimulus identity. (**a**) Evolution of odour-evoked ensemble responses over time is shown after dimensionality reduction using principal component analysis. The number of neurons (*n*) used for this analysis and the percentage of variance captured in the first three dimensions are shown in each plot. Numbers near response trajectories indicate time in seconds since odour onset. Red and blue trajectories are solitary introductions of two different odourants. Black trajectories reveal the ensemble responses following the introduction of the second odourant in the sequence. (**b**) Blue and red traces indicate pattern match between ensemble activities generated following the introduction of the second odourant with the response templates obtained for the component odourants (see Methods). (**c1**) Representative raster plots revealing responses of two different Kenyon cells in the insect mushroom body to odourants that do not produce coherent PN response during odour overlaps. (**c2**) Kenyon cell PSTHs (mean±s.d.; *n*=10 trials) are shown as a function of time for solitary (red) and overlapping (black) introductions of six different odourants (70 Kenyon cells in total; *P<0.01, paired *t*-test for peak firing rate comparison). Note that the six odourants used are those that evoked antennal lobe ensemble responses that were less synchronous when presented following another stimulus. (**d1**,** d2**) Similar plots as **c1**,** c2**, but analysing all Kenyon cell responses to those odourants that revealed coherent projection neuron responses no matter how they were introduced.

Does synchronization or desynchronization of ensemble neural responses alter the overall representation and therefore the encoded identity of the odourant? To understand this, we performed a qualitative dimensionality reduction analysis^3^,^12^ (Fig. 3a and Supplementary Fig. 7). We constructed a time-series of high-dimensional PN ensemble response vectors, where individual vector elements corresponded to spike counts of a single neuron in a given 50 ms time bin. These high-dimensional vectors were projected onto three dimensions using a linear principal component analysis technique. The low-dimensional points were connected in a temporal order to visualize the neural response trajectories.

We found that each odour evoked a closed loop trajectory in a certain direction. When presented following another stimulus, the responses following the introduction of the second stimulus in the sequence (black trajectories) were still aligned with the firing patterns elicited by solitary presentations of the same odour (red trajectories). However, for those odourants that had greater response overlaps with the preceding stimulus, the intensity of the response (that is, the length of the vectors) was substantially reduced. As expected, the length of the response vectors depended on whether the responses were synchronous or asynchronous across neurons. On the other hand, the response pattern match between solitary and overlapping presentations depended predominantly on whether the set of neurons activated (that is, the combinatorial code) remained consistent across presentation conditions (Supplementary Fig. 2e). We found that when the combinatorial code was altered significantly (for example, cit versus cit(ger) case; Supplementary Fig. 7), the pattern match also diminished across conditions. The qualitative dimensionality reduction results were quantified using a classification analysis (Fig. 3b and Supplementary Fig. 7; pattern matching done purely using vector similarity; see Methods). In sum, these results confirm that odour identity is robustly represented when only the temporal structure varies between ensemble responses elicited during isolated (synchronous response; Fig. 1a,b) and overlapping presentations (asynchronous response; Fig. 1c,d) of the same stimulus.

### Decoding different modes of antennal lobe responses

To understand how disruption in ensemble neural synchrony alters responses in the neural population downstream to the antennal lobe circuits, we monitored Kenyon cell responses in the insect mushroom body. We found that Kenyon cells were more likely to respond to an odourant during the periods of peak antennal lobe activity. Furthermore, they responded to an odourant irrespective of whether the odourant was delivered in isolation or succeeding another odourant (Fig. 3c1, d1, [Fig f1]; paired *t*-test, *P<0.01). However, the probability of response was significantly reduced for those odourants that failed to elicit a highly coherent antennal lobe response (Fig. 3c2, [Fig f2] versus Fig. 3d2, [Fig f2]; Supplementary Fig. 8). Hence, these results are consistent with the idea that synchronous neural inputs are likely to more reliably activate target cells^13^,^14^,^15^.

### Behavioural correlates of neural synchrony

**Figure 4:**
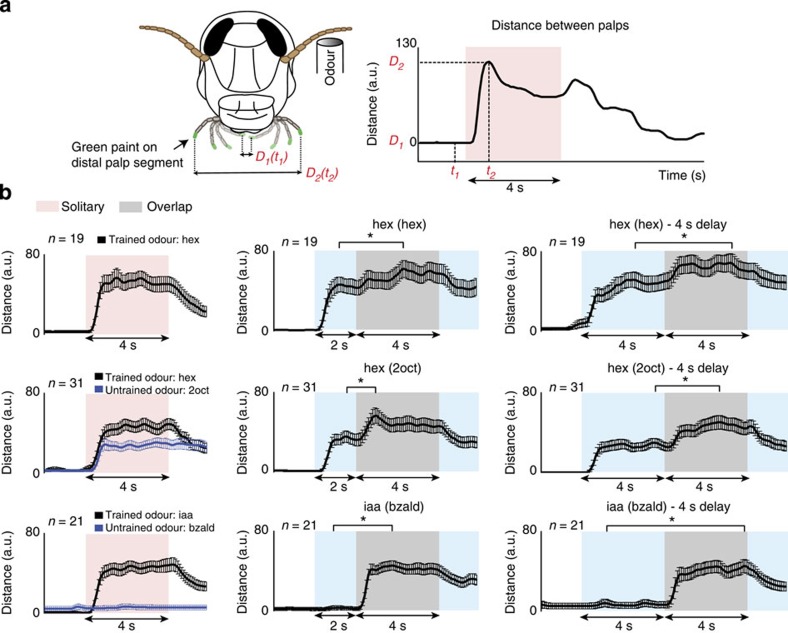
Behavioural responses to the conditioned stimulus are robust across presentation conditions. (**a**) A schematic of the locust palp-opening response (POR) assay. Opening of maxillary palps to the trained odourant was used as an indicator of acquired memory. The palps were painted with zero-volatile-organic-chemical green paint to facilitate tracking. The distance between the two palps on a frame-by-frame basis (100 ms temporal resolution) was calculated, smoothed and plotted as a function of time. (**b**) PORs tracked during test phase trials are shown for each type of stimulus presented (mean±s.e.m.). The conditioned stimuli (hex or iaa) were either presented alone (left column) or following another odourant with two different latencies: 2 s (middle column) or 4 s (right column). Responses to solitary presentations of the two untrained odourants (2oct and bzald) are plotted in blue in the leftmost column. Asterisks indicate significant increases in the peak POR elicited by the second stimulus (*P<0.05, Wilcoxon signed-rank test, *n* indicates the number of insects used in the test).

Finally, we examined whether the observed synchrony in ensemble neural response was relevant to odour-evoked behaviour in this model system. To investigate this, we trained locusts in an appetitive-conditioning assay^12^,^16^. During the training phase, an odourant (conditioned stimulus; hexanol or isoamyl acetate) was presented with a grass reward (unconditioned stimulus). Selective opening of the maxillary palps (sensory appendages close to the locust mouth) to the conditioned stimulus presentations in the unrewarded test trials served as an indicator of acquired memory (Fig. 4a).

To perform fine-grained analysis of the behavioural response, we painted both the palps and tracked their movement with 100 ms temporal resolution using a custom-written image processing software (Supplementary Movie 1). We found that trained locusts responded to the conditioned odour reliably and for the entire duration of the odour pulse (Fig. 4b; 1st column). The response onset was rapid and consistent across locusts (Median time=0.70±0.27 s for hexanol and 0.60±0.39 s for iaa; see Methods). We found that locusts trained with hexanol also responded to introductions of 2-octanol, an odourant that evoked a highly overlapping neural response (Fig. 4b; second row). Note, however, that the trained odour (hexanol) elicited a stronger response than the untrained odour (2-octanol). Conversely, locusts trained with isoamyl acetate, selectively responded to the trained stimulus alone and not to benzaldehyde introductions during the testing phase (Fig. 4b; bottom row).

We took advantage of our earlier results that revealed that locusts performed consistently when multiple unrewarded test trials were carried out in a back-to-back manner^12^. Matching our electrophysiology studies, we delivered pulses of a trained stimulus in a solitary or an overlapping manner. Pulses of the same or differing odourants (hex(hex), hex(2oct) or iaa(bzald)) with two different delays (2 and 4 s) were used to evaluate behavioural performance (Fig. 4b second and third columns). The 4-s delay was used to further facilitate decoupling of the behavioural responses elicited by the two stimuli delivered in quick succession.

We found that introductions of the trained stimulus (in isolation or in an overlapping sequence) elicited a distinct and significant palp-opening response (POR) in all cases (Fig. 4b; Wilcoxon signed-rank test, *P<0.05). Considering that these stimulus introductions elicited ensemble responses with completely different temporal structure (Fig. 1a–d), this result is consistent with our interpretation from physiology data that stimulus identity is robust to variations in the temporal structure of the ensemble activities.

**Figure 5:**
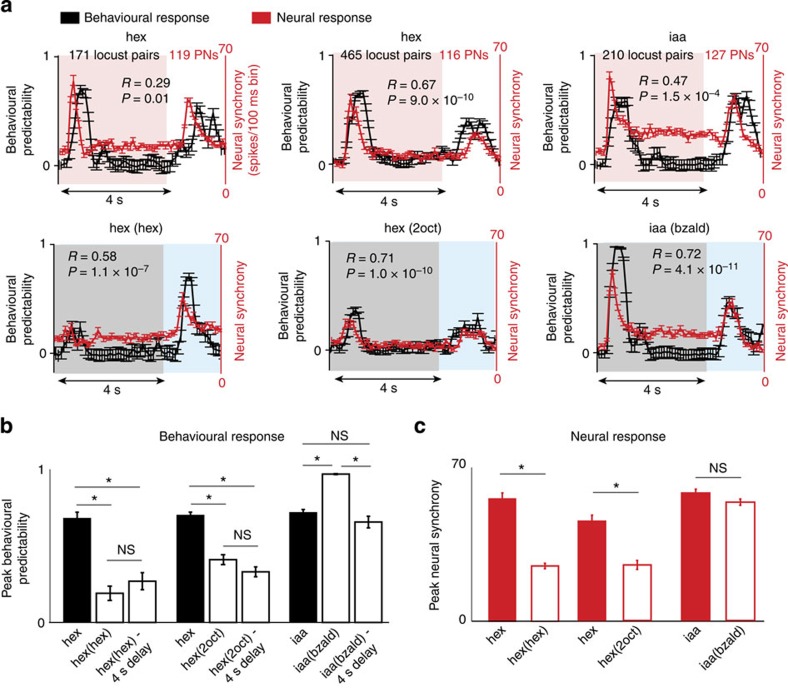
Behavioural response predictability correlates with ensemble neural synchrony. (**a**) Pairwise correlation between PORs (a measure of behaviour predictability) observed in different locusts were calculated as a function of time (mean±s.e.m.). The red traces quantify neural synchrony for the same set of odour stimuli (spike counts in 100 ms time bins were used to match the behavioural response resolution). Following conditioned stimulus introductions, significant correlations were observed between physiology and behavioural data for all conditions tested (Pearson's correlation coefficient; *n*=60 time bins including 4 s of odour exposures and 2 s of offset responses). (**b**) Maximum value of the pairwise correlation to solitary and overlapping presentations of different conditioned stimuli are summarized (mean±s.e.m.). Asterisks indicate significant change in pairwise correlation between PORs (*P <0.05, not significant (NS) is *P*>0.05; Wilcoxon signed-rank test with Bonferroni correction for multiple comparisons; *n*=171, 465, 210 pairs of locusts). (**c**) Comparison of the peak neural synchrony are shown for those stimuli also used in the POR assay (mean±s.e.m.; *n*=10 trials). Asterisks indicate significant reductions (*P<0.05, NS is *P*>0.05, paired *t*-test).

To understand how predictable the locust PORs were following introduction of the trained odourant, we computed the pairwise correlation between the behavioural responses observed in different locusts in a specific time segment (Fig. 5a and Supplementary Figure. 9; see Methods). The larger the pairwise correlation in a given time segment, the greater the predictability of the response based on responses observed in other locusts. Remarkably, a significant correlation was observed between the predictability of the behavioural responses to the conditioned stimulus and the neural response synchrony (as quantified by the summed spiking activity across neurons; Fig. 5a, black versus red traces, and Fig. 5b,c). Note that the behavioural responses of locusts were highly predictable at odour onsets and offsets for both conditioned stimuli. During middle portions of an odour stimulus or when responding to the second pulse of hexanol, the neural synchrony was diminished and the behavioural responses, although significantly above the baseline levels, were less predictable across locusts. Increasing the delay with which the second pulse of hexanol was introduced did not improve behavioural response predictability (Fig. 5b). Notably, for isoamyl acetate, the neural synchrony was not disrupted when presented in sequence with benzaldehyde. Correspondingly, the behavioural response was highly predictable for all isoamyl acetate introductions. Therefore, we conclude that the behavioural consequence of disrupting neural synchrony is not in eliminating the PORs altogether but in making them highly variable.

In sum, our results reveal a direct correlation between changes in neural synchrony and behavioural response predictability in the locust olfactory system.

### Combinatorial versus temporal odour code

Our physiology results revealed that the temporal structure of a stimulus could be disrupted by stimulus history. However, the odour identity was robustly represented by the ensemble neural responses. How is this achieved? To develop an intuition behind this neural computation, we developed a simple ensemble neural model. We modelled the response of each neuron to a stimulus using an exponential function with just two parameters: response amplitude and response time constant. Different stimuli were modelled to activate partially overlapping subsets of neurons (see Methods).

**Figure 6:**
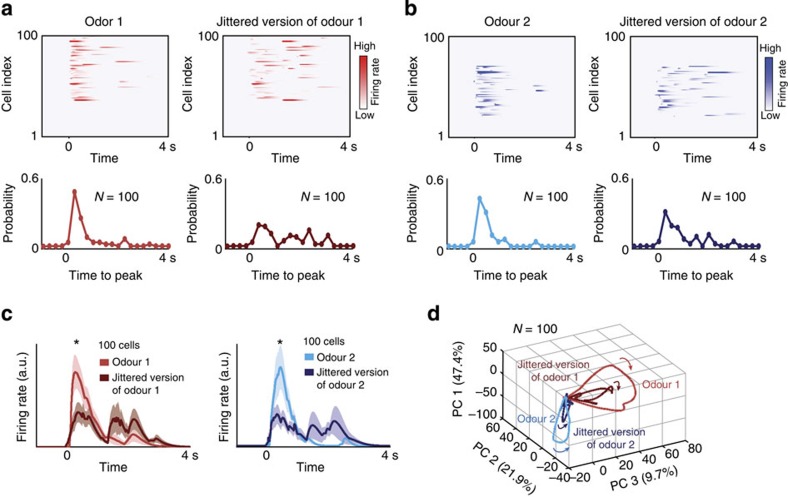
Evaluating combinatorial versus temporal properties of neural activity. (**a**) Simulated neural activities in a well-constrained computational model with 100 neurons are shown for a single odourant that activated the same set of neurons but with different temporal structures (left versus right panel). The fraction of neurons reaching their peak values at any given time period is shown below each image plot. (**b**) Similar plots as in **a** but now showing responses to a different simulated odourant. Note that the two odourants shown in **a** and **b** activate different but partially overlapping subsets of neurons. (**c**) The more synchronous the ensemble activity, the greater was the overall response as measured by the mean PSTH (±s.e.m.). Asterisks indicate significant difference in spike counts across neurons (*P<0.05, Wilcoxon signed-rank test, *n*=100). (**d**) A linear dimensionality reduction analysis of the simulated neural data revealed that odourants that activated the same set of neurons traced response trajectories that were aligned with each other irrespective of their differences in temporal structure.

We found that the disruption of temporal structure diminished the overall response intensity as shown by the response trajectories that dwarfed after the introduction of experimentally constrained variable delays (Fig. 6). However, both jittered and non-jittered versions of ensemble neural activities were still aligned and occupied the same subspace as shown by the dimensionality reduction analysis. On the other hand, a substantial change in the activated group of neurons resulted in a mismatch of population-level responses (refer to cit-ger trajectories, for example; Supplementary Figs 2d and 7). Hence, our results support the idea that as long as a conserved set of neurons is activated, the stimulus identity can be insensitive to variations in the temporal structure of their neural firing. This result also suggests that spatial (identity) and temporal (novelty, intensity) features of neural responses may be used for encoding non-redundant aspects of a stimulus.

## Discussion

In neural circuits, coordinated spiking activities across ensembles of neurons have been observed at a variety of temporal and spatial resolutions^17^,^18^,^19^,^20^,^21^,^22^,^23^,^24^,^25^. Such temporally precise responses have been shown to be important for learning^26^,^27^,^28^ and are thought to mediate various rhythmic field-potential activities observed at different oscillation frequencies in the brain^29^. However, whether synchronous neural activities facilitate different neural computations compared with asynchronous responses and whether different modes of neural transmission produce different types of behavioural responses remain unclear.

One prevalent hypothesis is that synchronous neural signals may provide a substrate to integrate pieces of information individually encoded by neurons (‘the neural binding' problem^30^), and are therefore considered important for neural computations^30^,^31^,^32^. This hypothesis, however, is still widely debated^33^,^34^,^35^. Furthermore, recent works have shown that stimulus-evoked neural activity can also be asynchronous in nature^36^,^37^,^38^,^39^, where temporal integration rather than coincidence detection has been proposed as a readout mechanism^36^. These observations further confound the functional role of coordinated spiking activity across neurons. Can a stimulus evoke both synchronous and asynchronous ensemble responses, and if so, when is a particular mode of neural activity preferred to transmit information? Our results describe an interesting mechanism based on synchrony that allows neural networks employing a combinatorial coding scheme to selectively de-emphasize certain sensory inputs. Notably, as shown in our computational model, desynchronizing ensemble activities only reduced the intensity of the circuit's response to the adapting and cross-adapting stimuli without altering their encoded identity.

We examined the relationship between spike synchrony with other forms of neural synchrony reported in these circuits. Previous studies have shown that odourants also entrain oscillatory synchronization of PN activities in the insect antennal lobe^2^,^20^,^40^,^41^,^42^. This neural synchronization resulted in field potential activity with power in the 20–30 Hz range and has been shown to slowly build up with repeated presentation of a stimulus^43^. Furthermore, abolishing the oscillatory activity in honey bees through pharmacological manipulation disrupted finer discrimination between odourants^20^. In contrast, in this work we revealed a non-oscillatory form of neural synchrony observed only at the stimulus onsets and offsets of a novel odourant. We note that these two forms of neural synchrony appear to only have a weak relationship with each other. For example, consistent with earlier work^20^,^40^, we found that local field potential (LFP) oscillations lasted the entire stimulus presentation duration even though the ensemble spike synchrony diminished within a second after odour onset. Conversely, LFP oscillatory synchronization was disrupted during stimulus offset but spike synchrony was high during these periods. Last, we found that overlapping sequences of stimuli only selectively disrupted ensemble spike synchrony, whereas the LFP oscillatory synchrony persisted (Supplementary Fig. 10; although the power in the 20–30Hz band reduced when spike synchrony was diminished for hex(2oct)). Integrating these results with our behavioural results we conclude that the behavioural response predictability correlated better with the non-oscillatory form of ensemble spike synchrony observed at odour onsets and offsets.

What are the possible mechanisms that might underlie the observed cross-adaptation between odourants? It is possible that some of our results may be explained by adaptation at the level of sensory neurons themselves. To investigate this issue, we analysed the sensory neuron responses to overlapping sequences of odourants. We found that the sensory neuron responses to the second odourant pulse reduced significantly only when the two pulses belonged to the same odourant (that is, hex(hex) case; Supplementary Fig. 11). When two different odourants were presented in a sequence, the response following the introduction of the second odourant was mostly comparable to those observed during its solitary presentation. Therefore, we speculate that dependence on stimulus history can arise either through reduction in synaptic efficacy between sensory neurons and their downstream PNs or by enhancing the inhibition to the co-active group of PNs by facilitating GABAergic inhibition through local neurons in a heterogeneous manner.

We found that both the overall combination of PNs activated as well as their activation profiles were relatively consistent across trials and across different presentation conditions (solitary versus overlapping), but varied considerably between odourants (Supplementary Fig. 2e). The temporal structure of responses, on the other hand, could vary depending on the presentation conditions (Fig. 1e and Supplementary Fig. 2c). Based on these results, two orthogonal predictions could be made. First, if the fidelity of the ensemble responses' temporal structure were important, then one possible outcome is that locusts will not recognize a trained odourant when it is presented in sequence following a ‘similar' odourant (here similarity refers purely to neural response overlaps, for example, hexanol and 2octanol are similar odours). Alternately, if the combinations of neurons activated alone were sufficient for odour recognition, then we would expect trained locusts to recognize a conditioned stimulus irrespective of whether it was presented solitarily or in a sequence. Our behavioural results are consistent with the latter hypothesis that a consistent combinatorial code may be sufficient for recognition of trained odourants by locusts (refer Fig. 4).

Interestingly, we found that the combinations of neurons activated by citral could change significantly depending on whether it was presented solitarily or in a sequence following geraniol (cit versus cit(ger) case; Supplementary Fig. 2e). This can be clearly seen in the mismatch between neural response trajectories traced by solitary and overlapping introductions of citral and further quantified in the classification analysis (Supplementary Fig. 7). Consistent with these results, in our earlier work^12^, we found that although solitary introduction of citral innately repelled locusts in a T-maze assay, when presented following geraniol with a 2-s lag, citral introductions failed to repel them from the arm delivering this overlapping odour sequence. Hence, these results suggest that odourant identity is altered when the combinatorial code is altered.

Furthermore, our results reveal that not all neurons that were activated by an odourant may contribute towards the onset of behavioural response. This is because we found that only a subset of PNs had response latencies less than 600 ms (median time required to open maxillary palps following the onset of conditioned stimulus in the behavioural assay; Supplementary Fig. 12). The spike synchrony across these ‘low-latency' neurons was in fact sufficient to explain the changes in the behavioural response predictability following odour onset in solitary and overlapping conditions (Supplementary Fig. 13). Hence, we expect these ‘low-latency' neurons to contribute significantly to encoding odour identity. We note that for all odourants used in this study, we also found a smaller subset of neurons with a delayed response (latency >600 ms). As odour recognition necessary to start the behavioural response in most trained locusts would have already happened before these neurons begin to respond, the contributions of these ‘high-latency' neurons to odour processing remains unclear.

We found several parallels between the temporal features of odour-evoked neural and behavioural responses. Consistent with previous works^4^,^9^,^10^,^11^, we found that odour-evoked responses were stronger and more dynamic immediately following odour onsets and offsets. In between these transient epochs of neural activity, a persistent stimulus could only evoke neural activity patterns that were less intense but more stable. Our dimensionality reduction and classification analyses results revealed that although the activity returned close to baseline levels during these steady-state epochs (causing a reduction in Euclidean distances between ensemble responses^9^), the high-dimensional response vectors were still aligned with those observed immediately following odour onsets (smaller angular distances between response vectors observed over time). Similarly, we found that the PORs of trained locusts to conditioned stimuli were rapid (Supplementary Fig. 12; median response latency 600–700 ms) and persisted as long as the overall population response in the antennal lobe was above the baseline level, that is, during both transient and steady-state periods. Furthermore, the behavioural response dynamics, albeit slightly delayed, still matched the ensemble neural response dynamics closely (Fig. 5). Finally, the behavioural responses across locusts were predictable only during those temporal epochs when the neural activities were highly synchronous.

Notably, we found that the behavioural consequence of disrupting synchrony was not in eliminating the responses altogether but in making them highly variable. Although a piecewise, high-threshold decoder would be appropriate for interpreting synchronous inputs, decoding an asynchronous response would involve a temporal integrator. Our results suggest that the circuits downstream to the insect antennal lobe may have the ability to interpret different modes of neural transmissions. It appears that temporal integration may happen as long as asynchronous ensemble neural activities still encode for the identity of the odourant, that is, neural responses trace trajectories within the ‘attractor' (or ‘sub-space') that encodes the stimulus identity.

## Methods

### Odour stimulation

Odour stimuli were delivered using a standard procedure as described in earlier works^6^,^12^. In brief, odour solutions were diluted in mineral oil to achieve 1% concentration (v/v) and placed in sealed glass bottles (60 ml) with an inlet and an outlet line. A pneumatic picopump (WPI Inc., PV-820) was used to displace a constant volume (0.1 l min^−1^) of the static headspace above the diluted odour solution into a desiccated carrier air stream (0.75 l min^−1^) that was directed at the locust antenna. A vacuum funnel placed right behind the locust preparation continuously removed the delivered odourants. Each odour stimulus was presented multiple times in one or two pseudorandomized blocks of five or ten trials each (five trials for sensory neuron recordings, ten trials for PN and Kenyon cell recordings). The inter-stimulus interval was at least 60 s for all recordings.

We re-analysed electrophysiology data from our recently published study^12^ for the following odour sequences: 2-octanol(2oct)–hexanol(hex), mint–apple, geraniol(ger)–citral(cit), hexanal(hxa)–hexanol(hex), benzaldehyde(bzald)–isoamyl acetate(iaa) and cyclohexanone(chex)–2-heptanone(2hep). In addition, the following new odour sequences were also collected to complete the neural data set: hexanol–hexanol, hexanol–2-octanol, hexanol–isoamyl acetate. Apple (apple green fragrant oil) and mint (peppermint supreme essential oil) were obtained from New Directions Aromatics Inc. All other odourants were obtained from Sigma-Aldrich.

### Electrophysiology

Young-adult (post-fifth instar) locusts (*Schistocerca americana*) of either sex were used for electrophysiological experiments. Extracellular recordings were performed from olfactory sensory neurons of different sensilla types using an intact but immobilized locust antennae as described previously^10^,^12^,^44^. Briefly, single sensillum recordings were performed by inserting saline-filled glass micropipettes (∼10 μm diameter, 5–10 MΩ) into the base of the sensory hairs or sensillum present on the surface of the locust antenna. An Ag/AgCl wire inserted into either the gut or the contralateral eye of the locust was used as the reference electrode. A differential amplifier (Grass P55) was used to acquire signals (filtered between 0.3 and 10.0 kHz) at sampling rate of 15 kHz (PCI-MIO-16E-4 DAQ cards; National Instruments).

To record odour-evoked extracellular neural responses from PNs in the antennal lobe and Kenyon cells in the mushroom body, locusts were immobilized with both antennae intact, and the brain was exposed, desheathed and superfused with locust saline at room temperature. For antennal lobe recordings, a 16-channel, 4 × 4 silicon probe (NeuroNexus) was used. Custom-made twisted wire tetrodes (Nickel Chromium wire, RO-800, Kanthal Precision Technology) were placed into superficial layers of the mushroom body for Kenyon cell recordings. A deeper placement of the tetrodes was preferred for LFP recordings. A panel of 1–3 odourants was used to prescreen a suitable mushroom body recording location that yielded at least one responsive Kenyon cell to any of the odourant in the panel. Multi-unit electrodes were electroplated with gold to obtain impedances in the range of 200–300 kΩ. Extracellular signals from PNs and Kenyon cells were recorded using a custom-made 16-channel amplifier (Biology Electronics Shop; Caltech). Signals were amplified at a 10-K gain, filtered between 0.3 and 6 kHz and sampled at 15 kHz using a LabView data acquisition system. A visual demonstration of these multiunit extracellular recording techniques is available online^44^.

### Spike sorting

To determine single-unit responses, spike sorting was done offline using the best three or four channels recorded and conservative statistical principles^45^. All PN and Kenyon cell single units were identified as described in our earlier work^12^. Briefly, to identify single units, the following criteria were used: cluster separation >5 noise s.d., number of spikes within 20 ms <6.5% and spike waveform variance <6.5 noise s.d.

For sensory neurons, data from a single extracellular electrode were used for spike sorting. Since, sensory neurons fired spikes rapidly and with extracellular action potential waveform that adapted in amplitude^10^,^12^, 20% of spikes were allowed to be within 20 ms of another spike and the spike waveform variance were ignored as long as the identified unit was well separated from others (that is, cluster separation >5 noise s.d. of all other spike waveform clusters). Using this approach, a total of 168 olfactory sensory neurons (from 34 locusts), 844 PNs (from 88 locusts) and 120 Kenyon cells (from 44 locusts) were identified.

### Responsive criterion for PNs and Kenyon cells

For a PN to be considered as ‘responsive' to an odour, the following two criteria had to be satisfied: (i) amplitude criterion: odour-evoked average (over trials) neural activity in at least one of the time bins during odour presentation window must exceed 6.5 s.d. of average baseline activity (response in a 2-s window before odour onset) and (ii) reliability criterion: the amplitude criterion has to be met in at least 50% of total trials. A Kenyon cell was considered ‘responsive' to an odour if it fired at least one spike during the odour presentation time period in four or more trials.

### Percentage of co-activation

Percentage of PNs that were activated by both odourants in an odour sequence was calculated using the following equation:


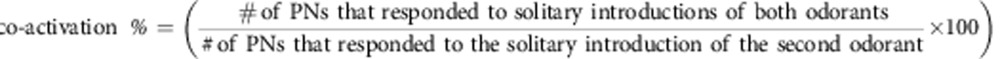


### Neural synchrony measure

Spike trains of PNs were segmented into non-overlapping time bins (1 and 50 ms in Supplementary Fig. 4, 10 ms in Fig. 1e, 100 ms in Fig. 5a). Spike counts within each time bin was summed across neurons and averaged over trials. For sensory neurons, a bin-size of 50 ms was used and for Kenyon cells a bin-size of 100 ms was used. A three-point average zero-phase digital filter was used to smooth the raw spike counts computed.

### Response latency

We conservatively defined response latency of a neuron as the time taken for the stimulus-evoked activity to reach 20% of the peak firing rate (bin size=50 ms). To account for variations in the spontaneous firing rates that might otherwise influence the response latency calculations, the stimulus-evoked firing rate responses were standardized first by subtracting the mean spontaneous activity observed during a 2-s pre-stimulus period. The same criterion was also used for determining the latency of a POR in our behavioural assay.

### Correlation of ensemble PN response profiles

For each PN and for each trial, spike counts were summed over the entire 4 s odour presentation period following the solitary introduction of an odourant or following the introduction of the second stimulus in the overlapping sequences. For a particular trial, this resulted in *n* spike counts for *n* PNs in the data set. The different correlation values shown in Fig. 1f and Supplementary Fig. 2d,e were computed as follows:

1. PN spike count profiles obtained during different trials (solitary introductions) were correlated in pairwise manner (inter-trial correlation).
2. Correlations between trial-averaged response profiles generated during solitary versus overlapping condition (inter-condition correlation).
3. Correlations between trial-averaged response profiles generated during solitary introductions of two different odourants presented in an overlapping sequence (inter-odour correlation).

A two-way ANOVA with bonferroni correction was used for the statistical testing. No significant difference was observed between the inter-trial group and inter-condition group (*P*=0.12). However, inter-odour correlation values were significantly lower than the inter-trial group (*P*=1.53 × 10^−5^) and the inter-condition group (*P*=7.25 × 10^−4^). Moreover, no significant differences were discerned between different odour groups (*P*=0.58).

### Spike counts comparison of individual PNs

Spike counts for cells determined to be ‘responsive' (see above) were computed over the entire 4 s odour duration following solitary introduction and following the introduction of the same odourant in the overlapping sequences. Comparisons between spike counts were made between these two conditions (solitary versus overlapping) for each PN using paired *t*-test at *P<*0.05 significance level.

### Dimensionality reduction analysis

For this analysis, we first arranged the ensemble PN responses as time series data of *n* dimensions (where *n* is number of neurons recorded) and *m* steps (the number of 50 ms time bins, where *m*=80). Only 4 s of PN activity during the solitary odour introductions and the neural responses following the introduction of the second odourant in the overlapping sequence were used for this analysis. Each ensemble PN response vector (in a given 50 ms time bin) was projected onto the three eigen vectors of the response covariance matrix that accounted for the most variance in the data set. Finally, the low-dimensional points were connected in a temporal order to visualize neural response trajectories to different stimuli. Plots shown in Fig. 3 and Supplementary Fig. 7 were generated after smoothing with a three-point running average low-pass filter.

### Classification analysis

We used the same classification algorithm that we proposed in our earlier study^12^. Briefly, ensemble response vectors obtained during solitary exposures of an odour were regarded as the desired reference templates to be pattern matched. Five trial-averaged reference templates were generated for each odour. These reference templates represented the mean ensemble PN activity during four 1 s temporal segments following odour onset (0–1 s, 1–2 s, 2–3 s, 3–4 s) and a 2-s window following odour offset.

For classifying trials that involved solitary odour presentations, we followed the leave-one-trial-out validation approach. In other words, nine trials were used as training trials for constructing the reference templates and the excluded trial became the test trial. This was repeated ten times such that each of the ten trials was made a test trial once. For overlapping conditions, all ten trials were regarded as test vectors to be classified using templates obtained from solitary odour exposures.

To identify meaningful response patterns, we defined a tolerance threshold that would restrict the classification analysis to include only those vectors that are within a certain angular distance (85° angular distance threshold used for all classification analyses) to any one of the desired response templates. Angular distances between a given test vector (**V**_**t**_) and each reference vector (**V**_**r**_) were computed as follows:


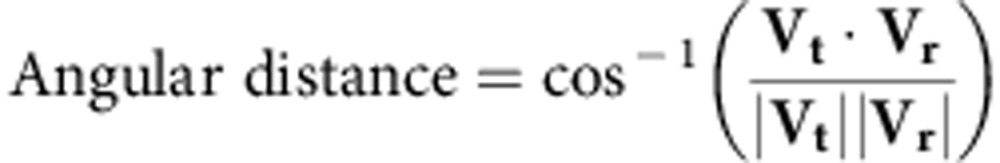


Only those test vectors that were within the defined tolerance threshold were classified. Note that no vector-length thresholding was done to limit the classification analysis to odour onsets and offsets when comparatively stronger responses were observed.

### LFP analysis

LFP signals were acquired at 15 kHz and filtered using an analogue filter (3–1,000 Hz). The raw data were subsequently re-sampled at 1 kHz and digitally filtered (10–50 Hz, 2nd-order Butterworth). Spectrograms were computed using a 500-ms sliding Hamming window with 90% overlap and averaged across five experiments with ten trials each. To allow comparison, power spectra computed for each trial were normalized by the maximum value in that trial. Cross-correlograms between signals simultaneously acquired from two electrodes were calculated using a 250-ms non-overlapping time windows and averaged across ten trials.

### Behaviour experiments

An appetitive palp-opening paradigm was used for behavioural experiments reported here. Adult locusts of either sex were starved for 24 h before the experiments. Locusts were immobilized within a plastic tube such that the antenna and mouthparts were freely movable. Both compound eyes were closed using black electrical tape to reduce the influence of visual cues on behaviour and reduce spontaneous activity. To precisely track the palp movements, a small amount of zero-volatile-organic-chemical green paint (Valspar Ultra) was applied to the distal segments of both the maxillary palps covering approximately 1 mm of the length of the palp. The painting of palps was done approximately an hour before the experiments for the locusts to become acclimatized with their coated palps and for the paint to dry.

For all experiments, we used either hexanol or isoamyl acetate as the conditioned stimulus. Wheat grass was used as the unconditioned stimulus. The odour delivery setup was identical to that in our electrophysiology experiments. A video camera (Microsoft webcam) was used to capture the behavioural responses of locusts during the training and test trials at ten frames per second (that is, 100 ms time resolution). A custom-written Labview programme controlled both the odour delivery and video data acquisition and thereby ensured a tight time match between them.

Six trials were used to train each locust to associate the conditioned stimulus with the food reward. During each training trial, a 10-s conditioned stimulus was presented first, which was followed by a grass reward that was offered 4 s after conditioned stimulus onset. The inter-trial interval was set to 10 min. To exclude any preconditioning to the conditioned stimulus, locusts that responded to the conditioned stimulus in the first training trial were eliminated from further experiments (<14%). Only those locusts that accepted the reward in at least four out of six training trials were used for testing (>85%).

During the testing phase, we presented the conditioned stimulus or an untrained odourant (2-octanol or benzaldehyde) solitarily or in an overlapping sequence in a random order. In all such odour sequences, the conditioned stimulus was always presented as the second stimulus with an onset delay of 2 and 4 s (with respect to the onset of the first stimulus in the sequence).

Locusts were kept on a 12-h day–12-h night cycle (0700–1900 hours day). All behavioural experiments were performed between 0900 and 1500 hours.

### Palp-tracking algorithm

To develop a quantitative, fine-grained behavioural analysis, we tracked the locust palp movements using a custom-made Matlab programme offline. Briefly, each video file was converted into a series of RGB colour frames. After subtracting the greyscale image from the green channel of each frame, a 2-D Gaussian filter (10 pixel by 10 pixel; s.d. 10 pixels) was applied to emphasize the intensity of the green palps. The adjusted image was subsequently thresholded and filtered to generate a final image that contained only the painted segments of the maxillary palps. The centroids of the palps were tracked in all frames. One or both the palps were sometimes blocked from view due to movements of the antennae or poor video focus on the palps. In such cases, the palp positions were estimated from the previous frame. The precision of this palp tracking approach was subsequently visually inspected and validated on every single video. Four out of seventy-five locusts whose palps could not be tracked in this automated manner were eliminated from the data set. Even if the tracking failed in only one test trial, all data from those locusts were removed from further analysis. A demonstration of this approach is shown in Supplementary Movie 1.

### Analysis of behavioural data

There was very little palp activity during the pre-stimulus baseline periods on most trials. Therefore, even a small movement of palps that exceeded 20 pixels distance during single odour presentation was counted as response to the presented odourant. Distances between two palps were smoothed using a three-point zero-phase digital filter. The behavioural response latency was defined as the time when the POR distance reached 20% of its maximum value during trained odour presentation.

The resting palp positions were either fully closed or slightly open. To remove this variability in baseline palp separation, we calculated the resting distance between the palps 10 s before the onset of the odourant and subtracted this distance from all other calculated distances.

### Pairwise behavioural response correlations

Cross-correlations between palp movements of different locusts were calculated by first segmenting the entire behavioural response into overlapping 500 ms time segments (80% overlap between consecutive segments). A small amount of Gaussian noise (zero mean and 0.01 s.d.) was added to the calculated palp distances to eliminate infinite correlation values during time periods when the palps were held still. Note that this was the measure of predictability used in the analysis reported in Fig. 5a and Supplementary Fig. 9.

### Computational model

We simulated an ensemble of 100 PNs where each individual PN's firing activity was modelled as an alpha function:


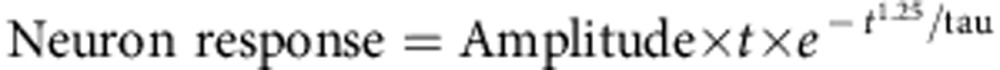


where Amplitude is the peak response amplitude, *t* represents the time and tau is the response time constant. The response amplitude for each cell was generated randomly from an exponential distribution with mean value of 10 Hz. The time constant for each cell was chosen randomly from a uniform distribution between 0 and 1 s. Ensemble responses to two different odourants (odour1 and odour2) were simulated by using two different sets of randomly chosen Amplitude and tau values. Furthermore, we experimentally constrained the model as follows: 65% neurons were activated by odour1 (corresponding to hexanol), 50% were activated by odour2 (corresponding to 2-octanol), and the degree of response overlap was obtained from Fig. 2d (2oct(hex) odour pair).

To simulate jittered versions of each odour, response delays were bootstrapped from the time-to-peak distributions (Supplementary Fig. 3) derived from solitary and overlapping stimulus presentations of hex(hex) and 2oct(hex) odour pairs (for odour1 and odour2, respectively).

A Matlab implementation of this model can be accessed at http://labs.seas.wustl.edu/bme/raman/publications/ncomm/ncmodel.m

### Justification of statistical tests

All statistical significance tests done in the manuscript were two sided. Bonferroni-corrected *P* values were used for all multiple comparisons. No statistical methods were used to predetermine sample sizes, but our sample sizes are similar to those reported in previous publications in the field^3^,^8^,^9^,^12^.

Levene's test was used to assess the homogeneity of variance between *time-to-peak-response* distributions (Supplementary Fig. 3). No normality test was performed because this test is suitable for non-normally distributed data as well.

For the paired *t*-tests and two-way ANOVA, normality of the data set was confirmed using the Jarque-Bera test. The equal variance assumption was tested using the Levene's test (Figs 1e,f inset, 3c2,3d2,5c, and Supplementary Figs 2c,2d inset, 2e,4b,6 and 11). The confidence level was set to 0.05 for both tests. In addition, for two-way ANOVA tests, the observations within and between groups were assumed to be independent (Supplementary Figs 2e and 6b).

Wilcoxon signed-rank test is a non-parametric test for comparing the population median responses of matched samples. This test was used to detect whether a significant increase in PORs was elicited by the second stimulus in the sequence (Fig. 4b), and to assess whether the predictability of PORs on the same set of locusts differed depending on how the conditioned stimulus was presented (Fig. 5b).

The Wilcoxon rank-sum test was used to compare the median population response for non-paired samples. We used this test to determine whether the neural and behavioural response latency distributions between odourants, that is, the comparison between hexanol and isoamyl acetate responses, were significantly different (Supplementary Fig. 12).

Linear regression analysis assumes that data can be fit using a straight line and sample points are independent of each other. Both of these assumptions were met in our analysis to detect the relationship between neural response overlaps and reduction in synchrony (Fig. 2e). Pearson's correlation was used to determine the degree of linear relationship between neural synchrony and behavioural response predictability (Fig. 5a). A linear relationship between the variables examined was assumed.

## Author contributions

B.R. conceived the study and designed the experiments. D.S., C.L. and N.K. performed the electrophysiological recordings. S.P. did the behavioural experiments. W.P. developed the palp-tracking algorithm. C.L. and D.S. analysed the data. B.R. and C.L. performed the modelling work. B.R. wrote the paper, and D.S., C.L., N.K. and W.P. provided feedback on the manuscript. S.P. and W.P. are equally contributing second authors.

## Additional information

**How to cite this article:** Saha, D. *et al.* Behavioural correlates of combinatorial versus temporal features of odour codes. *Nat. Commun.* 6:6953 doi: 10.1038/ncomms7953 (2015).

## Supplementary Material

Supplementary Information Supplementary Figures 1-13 and Supplementary Table 1

Supplementary Movie 1 Fine-grained tracking of locust palp movements in an appetitiveconditioned behavioral assay: (a) Palp-opening response to a conditioned stimulus (hexanol in this case) is shown in the top left panel. The green boxes indicate tracked palp locations. The entire video is 12 seconds long and contains the following segments: a 5 seconds pre-stimulus baseline period, followed by a 4 seconds exposure of hexanol, and finally a 3 seconds post stimulus period. (b) A filtered version of the same video using a custom-written image processing software is shown. The two blobs of white pixels identify the location of the two palps in each video frame. (c) The centroids of the two blobs are plotted as a function of time. Centroid locations in consecutive frames are linked using a line to show the evolution of the behavioral response. (d) Distance between the two tracked palps is shown over time. The y-axis corresponds to the number of pixels between the centroids of the two palps (arbitrary units). The colored box identifies the odor presentation period. Note that all four panels are synchronized in time.
