# Supplementary material for: Behavioural correlates of combinatorial versus temporal features of odour codes
Source: Nat Commun. 2015 Apr 27;6:6953. doi: 10.1038/ncomms7953 (PMC4421803; doi:10.1038/ncomms7953)
Supplement: Supplementary Information — Supplementary Figures 1-13 and Supplementary Table 1 [file ncomms7953-s1.pdf]

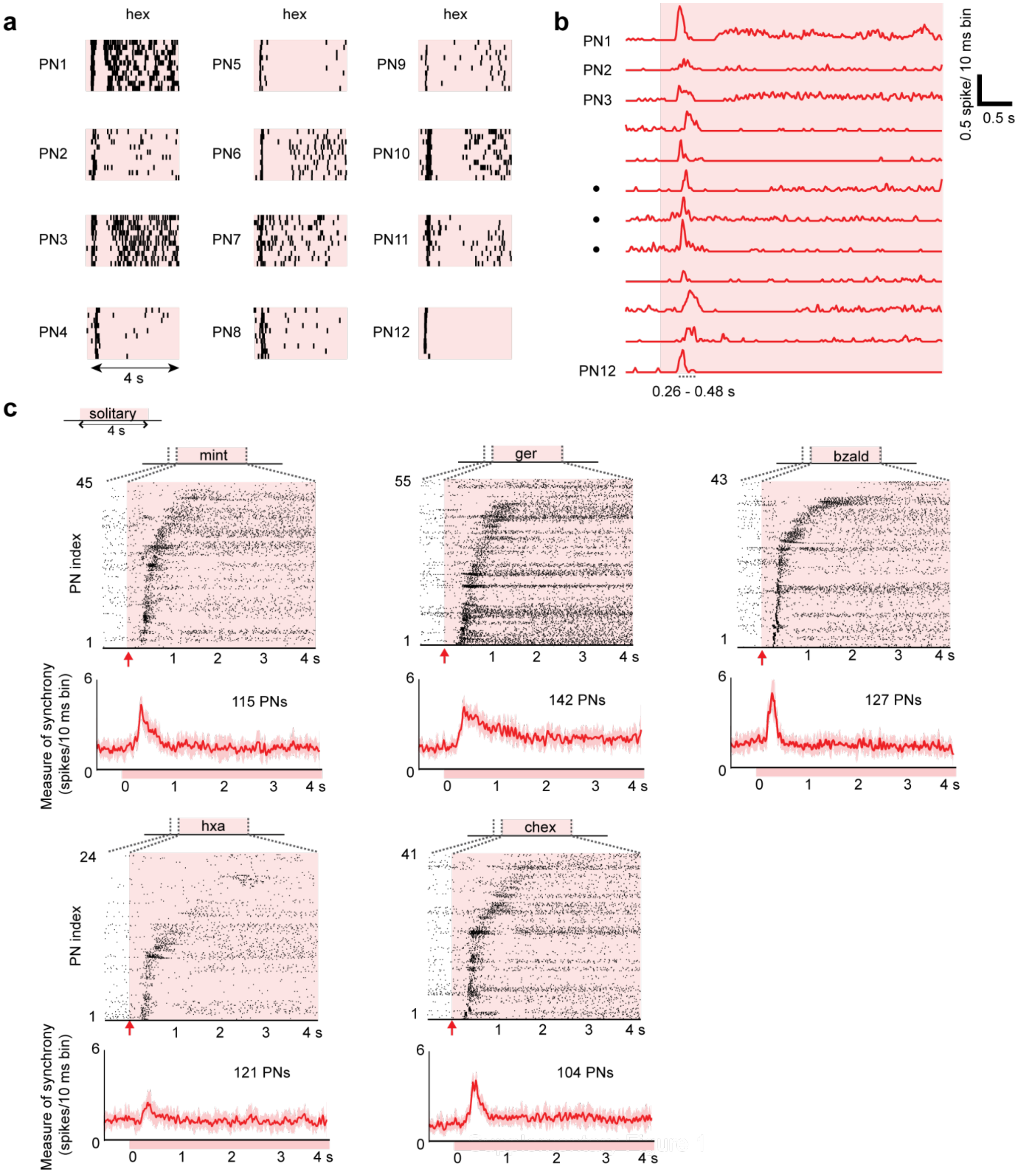

Supplementary Figure 1. Solitary presentation of odorants evokes synchronous spiking activities in the antennal lobe. (a) Responses of twelve different projection neurons (PNs) to a single odorant (hexanol) are shown as raster plots (10 trials each). The colored box indicates the four seconds of odor exposure. **(b)** Stacked peristimulus time histogram (PSTH) of each PN in **panel a** is shown. A 220 ms time window within which all twelve neurons reached their peak firing rates is identified along the x-axis. **(c)** Similar plots as in **Fig. 1b** but now showing ensemble PN responses evoked by other odorants used in the study: mint, geraniol (ger), benzaldehyde (bzald), hexanal (hxa), and cyclohexanone (chex).

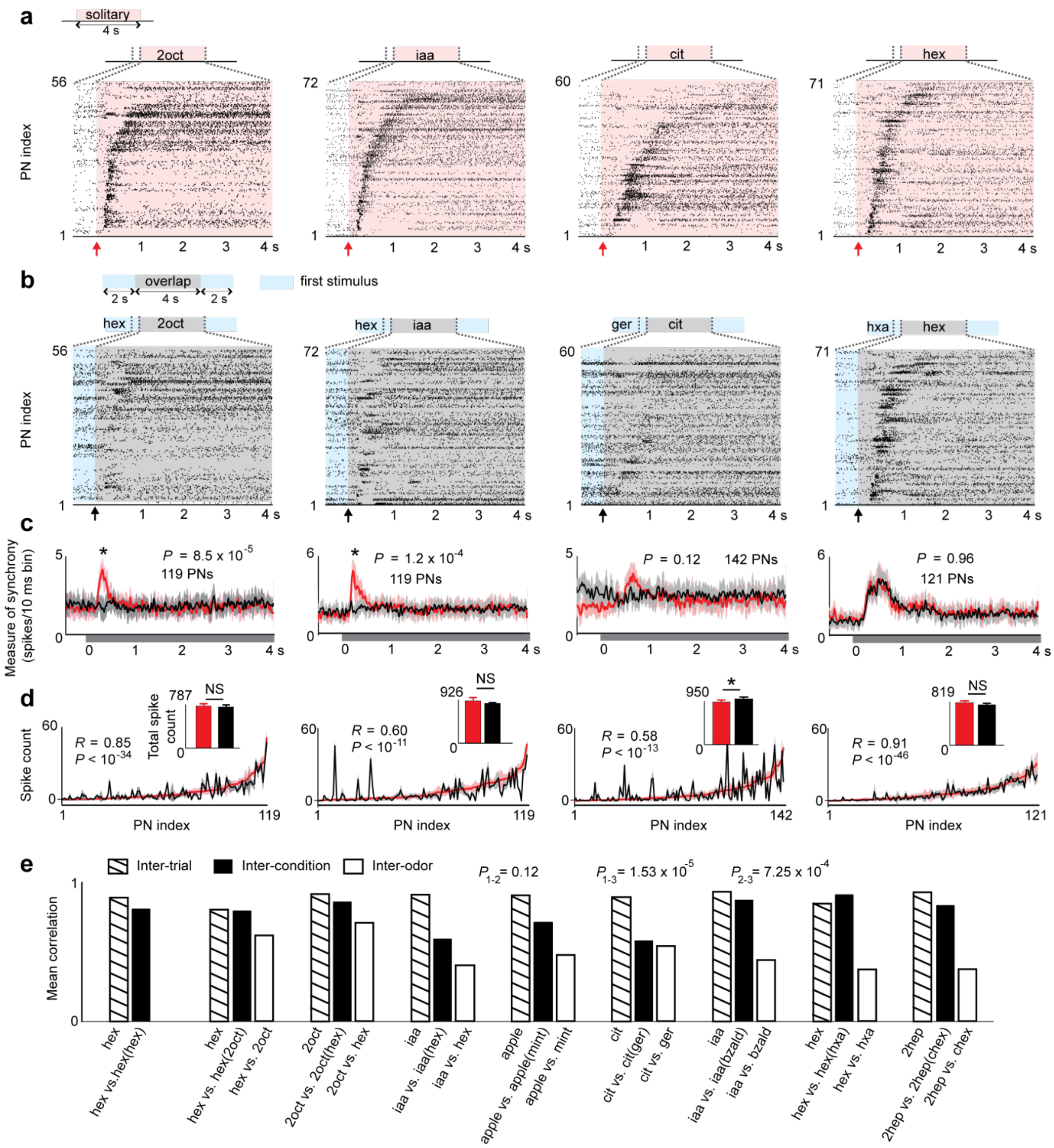

Supplementary Figure 2. Odorants can evoke asynchronous spiking activities in the antennal lobe depending on stimulus history. (a-d) Similar plots as shown in **Fig. 1b,d-f** but showing responses to the other overlapping sequences used in this study. Note that electrophysiology data for each odor sequence were collected on different sets of locusts (with some overlaps). **(e)** Correlation between spike-count profiles across projection neurons (see Methods) are shown for: (i) inter-trial responses (striped bars), (ii) inter-condition responses (solitary vs. overlapping introductions; solid bars), and (iii) inter-odor responses (open bars). A two-way ANOVA analysis involving all 8 odor-pairs (except hex vs. hex(hex) case), indicate significant changes in correlation were only observed between response profiles elicited by different odorants (Two-way ANOVA, *P(inter-trial vs. inter-condition)* = 0.12 (Bonferroni corrected for multiple comparisons), *P(inter-trial vs. inter-odor)* = 1.53×10^{-5}, *P(inter-conditions vs. inter-odor)* = 7.25×10^{-4}).

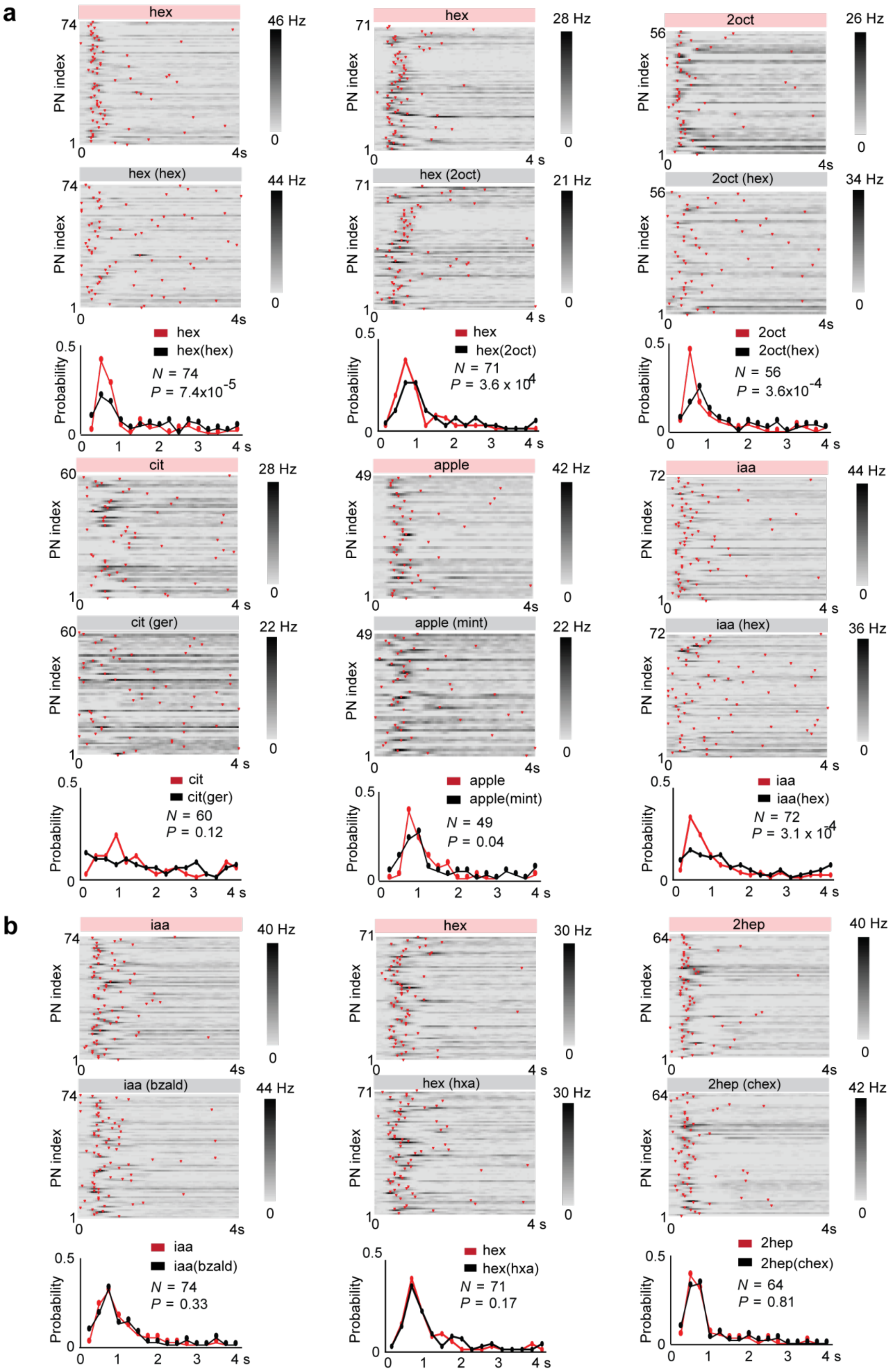
a
hex
hex (hex)
hex
hex(hex)
N = 74
P = 7.4x10-5
hex
hex (2oct)
hex
hex(2oct)
N = 71
P = 3.6 x 10-4
2oct
2oct (hex)
2oct
2oct(hex)
N = 56
P = 3.6x10-4
cit
cit (ger)
cit
cit(ger)
N = 60
P = 0.12
apple
apple (mint)
apple
apple(mint)
N = 49
P = 0.04
iaa
iaa (hex)
iaa
iaa(hex)
N = 72
P = 3.1 x 10-4
b
iaa
iaa (bzald)
iaa
iaa(bzald)
N = 74
P = 0.33
hex
hex (hxa)
hex
hex(hxa)
N = 71
P = 0.17
2hep
2hep (chex)
2hep
2hep(chex)
N = 64
P = 0.81
PN index
Probability

Supplementary Figure 3. Altering stimulus history can disrupt temporal coherence of ensemble neural activity. (a) PN ensemble responses to solitary and overlapping presentations of six odors are shown. Peristimulus time histograms (PSTHs) were averaged over trials and are shown as gray scale images for responsive projection neurons (see Methods). Each row reveals the response of a single projection neuron during the four seconds of odor exposure (80 time bins). Red triangles mark the peak of each projection neuron PSTH. Vertical alignment of the red triangle markers indicates that a large fraction of projection neurons reach their peak response in a highly coherent manner for all solitary odors introductions. For comparison, the bottom panels show ensemble responses to the same set of odorants but presented in an overlapping sequence with another odorant (projection neuron ordering is same in both panels). Note that the peak of the PSTH response to the second stimulus in the sequence happens with different latencies across the ensemble. The fraction of PNs reaching their peak value in a given 250 ms time bin is shown for different odorants (*time-to-peak-response* distribution). The *time-to-peak-response* distribution for solitary introductions is shown in red and the response distribution for overlapping odor introductions is shown in black. Significant differences in *time-to-peak-response* distribution variances were determined using Levene's test. (**b**). Similar plots as in **panel a**, but showing responses of three odorants that generated synchronous activity when delivered solitarily or in an overlapping fashion.

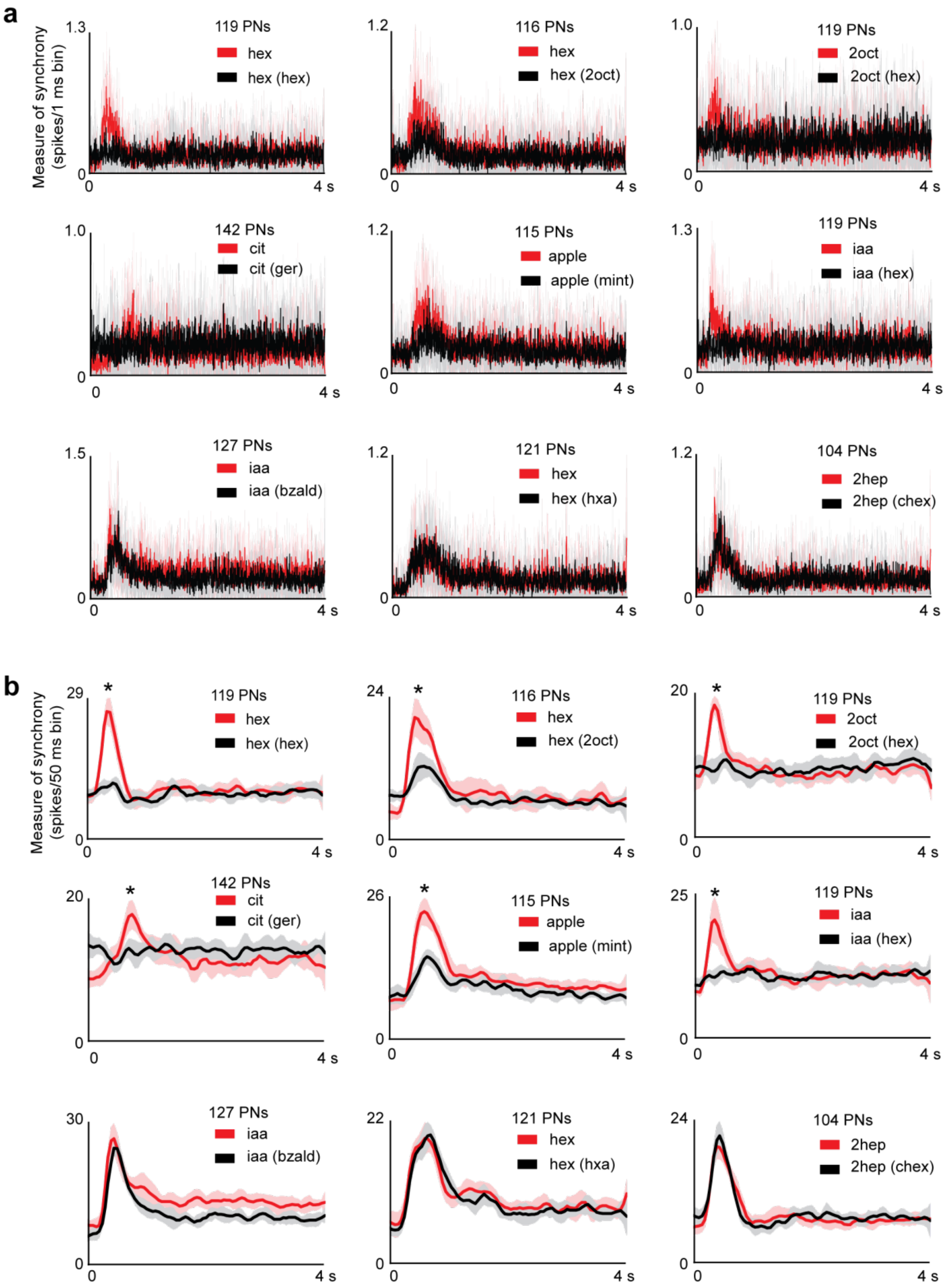

Supplementary Figure 4. Synchronous projection neurons activity can be identified independently of the time bin size. (a) Population projection neuron PSTHs are shown for 1 ms time bins for both solitary (red) and overlapping stimulus presentations (black). Number of PNs for each odor pair is shown in each panel. **(b)** Similar plots with 50 ms bin size. A paired t-test was used to detect a significant change in the peak spike count values (*$P < 0.01$, $n = 10$ trials).

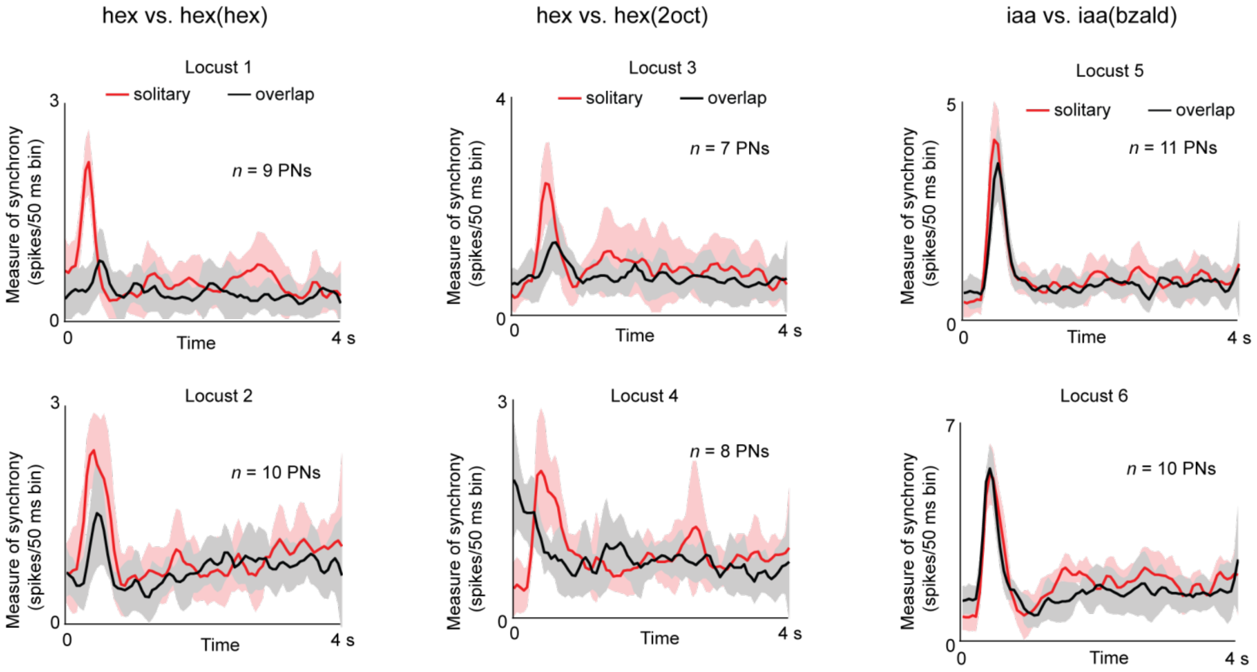

Supplementary Figure 5. Qualitatively similar results obtained by analyzing projection neurons simultaneously recorded from a single locust. Summed spike counts are shown but computed only using projection neurons recorded simultaneously from a single locust. Consistent with other plots, spike counts following solitary introductions are shown in red, and following overlapped introductions are shown in black. Note that the odorants and the sequences are the same as those used in the behavioral experiments. Results from two different locusts for each stimulus/stimulus sequence are shown for repeatability of the observed results.

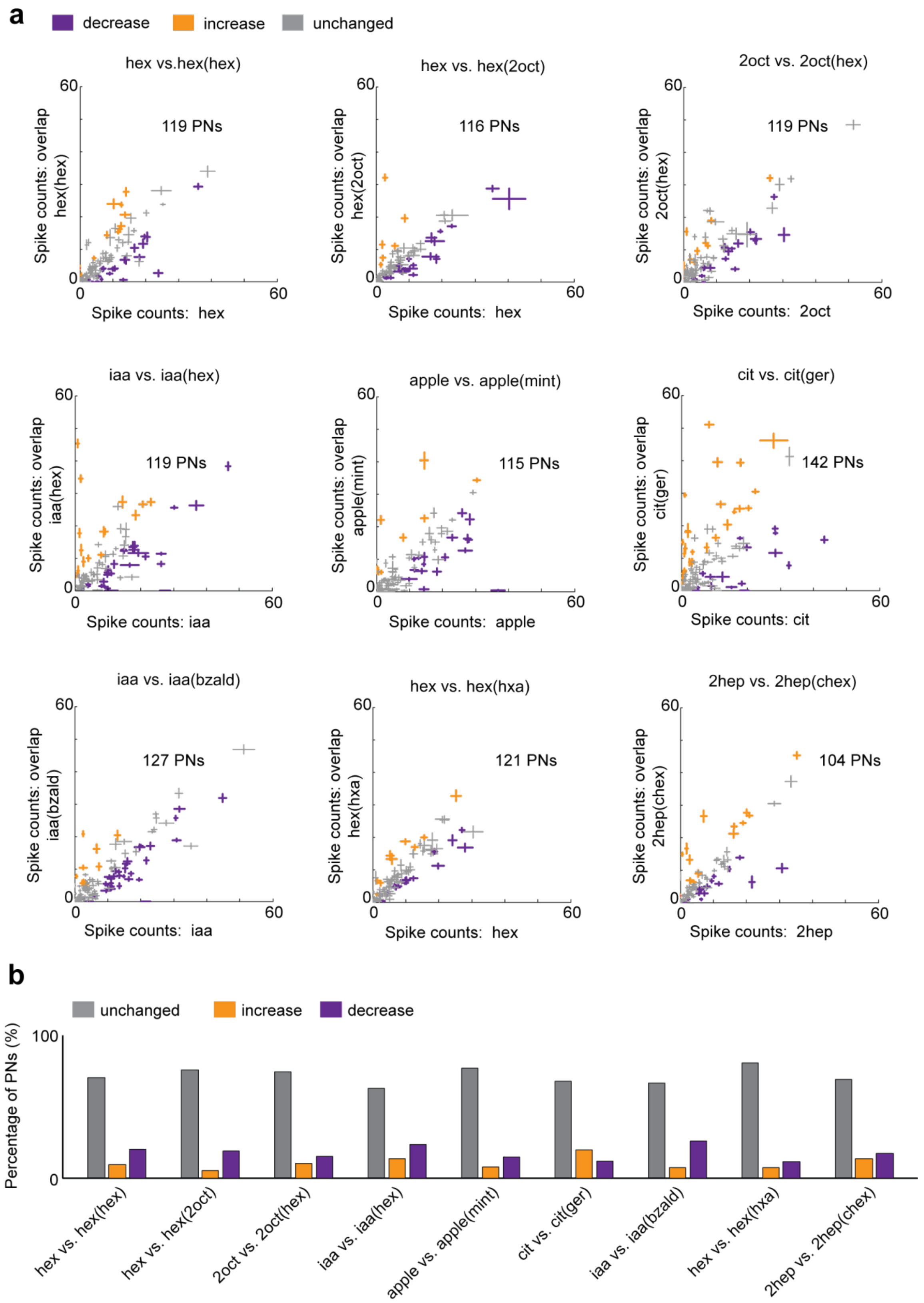

a
decrease
increase
unchanged
hex vs.hex(hex)
119 PNs
Spike counts: overlap hex(hex)
Spike counts: hex
hex vs. hex(2oct)
116 PNs
Spike counts: overlap hex(2oct)
Spike counts: hex
2oct vs. 2oct(hex)
119 PNs
Spike counts: overlap 2oct(hex)
Spike counts: 2oct
iaa vs. iaa(hex)
119 PNs
Spike counts: overlap iaa(hex)
Spike counts: iaa
apple vs. apple(mint)
115 PNs
Spike counts: overlap apple(mint)
Spike counts: apple
cit vs. cit(ger)
142 PNs
Spike counts: overlap cit(ger)
Spike counts: cit
iaa vs. iaa(bzald)
127 PNs
Spike counts: overlap iaa(bzald)
Spike counts: iaa
hex vs. hex(hxa)
121 PNs
Spike counts: overlap hex(hxa)
Spike counts: hex
2hep vs. 2hep(chex)
104 PNs
Spike counts: overlap 2hep(chex)
Spike counts: 2hep
0
60
b
unchanged
increase
decrease
Percentage of PNs (%)
100
0
hex vs. hex(hex)
hex vs. hex(2oct)
2oct vs. 2oct(hex)
iaa vs. iaa(hex)
apple vs. apple(mint)
cit vs. cit(ger)
iaa vs. iaa(bzald)
hex vs. hex(hxa)
2hep vs. 2hep(chex)

Supplementary Figure 6. Spike count analysis of individual projection neurons. (a) Comparison of mean projection neuron spike counts in a 4 s window after solitary introduction of an odorant or following the introduction of the same odorant as the second stimulus in an overlapping sequence. The *x* axis corresponds to spike counts when the odor is presented alone. The *y* axis corresponds to spike counts when the odor is presented after a preceding stimulus. The mean ± s.e.m. over ten trials is shown for all cells. Cells in orange indicate a significant increase in spike counts during the overlapping conditions and are therefore located above the diagonal ($P < 0.05$, paired t-test). Similarly, cells in purple indicate a significant decrease in spike counts, and cells in gray indicate no significant change across the two conditions. **(b)** Bar plot showing the number of cells with significant increase, decrease or no change across presentation conditions for the different odorants used in the study. No significant difference was observed between distributions computed for different odor groups (two-way ANOVA; $P = 1.0$).

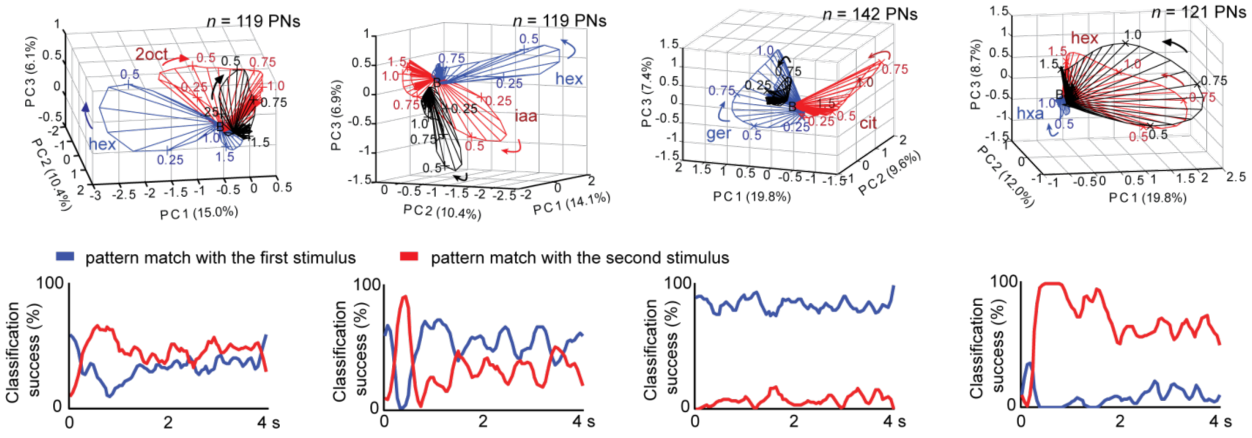

Supplementary Figure 7. Visualizing synchronous and asynchronous ensemble activities. Similar plots as in **Fig. 3a, b** but characterizing ensemble activity evoked by the four other odor pairs used in the study: 2oct(hex), iaa(hex), cit(ger), and hex(hxa). Note that the stimulus identity is robustly maintained for all overlapping cases except cit(ger).

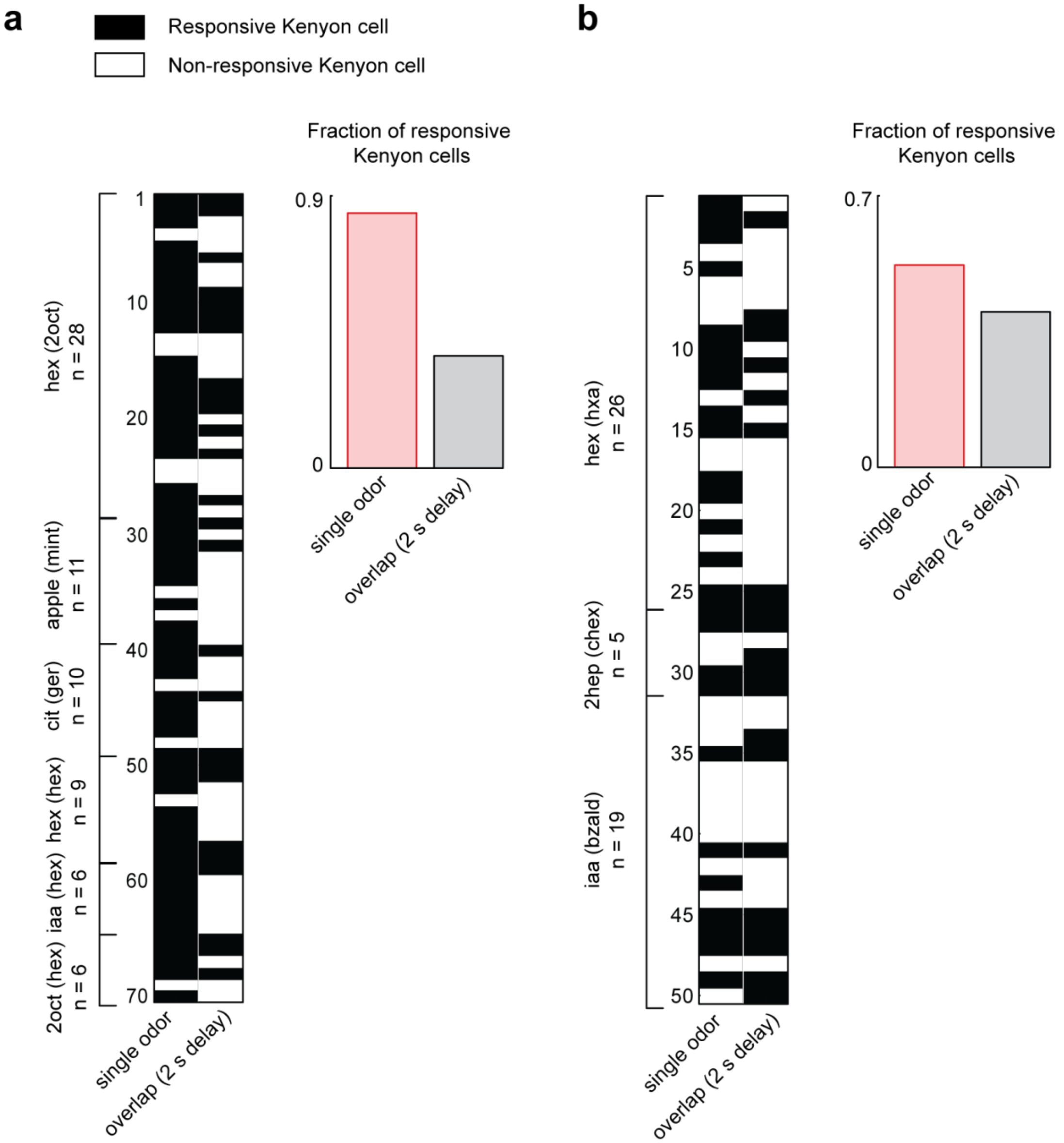

Supplementary Figure 8. Kenyon cell spiking probabilities to solitary and overlapping presentations of a panel of odorants. (a) Left, response of each Kenyon cell (KC) is shown for single and overlapping presentations of different odorants. Black represents 'responsive' and white represents 'non-responsive' KCs (see Methods). Right, fraction of KCs that responded to different introductions of the same set of odorants is summarized. All KCs recorded for odor pairs that elicited asynchronous PN ensemble activity in the antennal lobe were combined for this analysis. **(b)** Similar plots as in **panel a** but characterizing Kenyon cell responses to those odorants that had coherent projection neuron responses to both solitary and overlapping presentations.

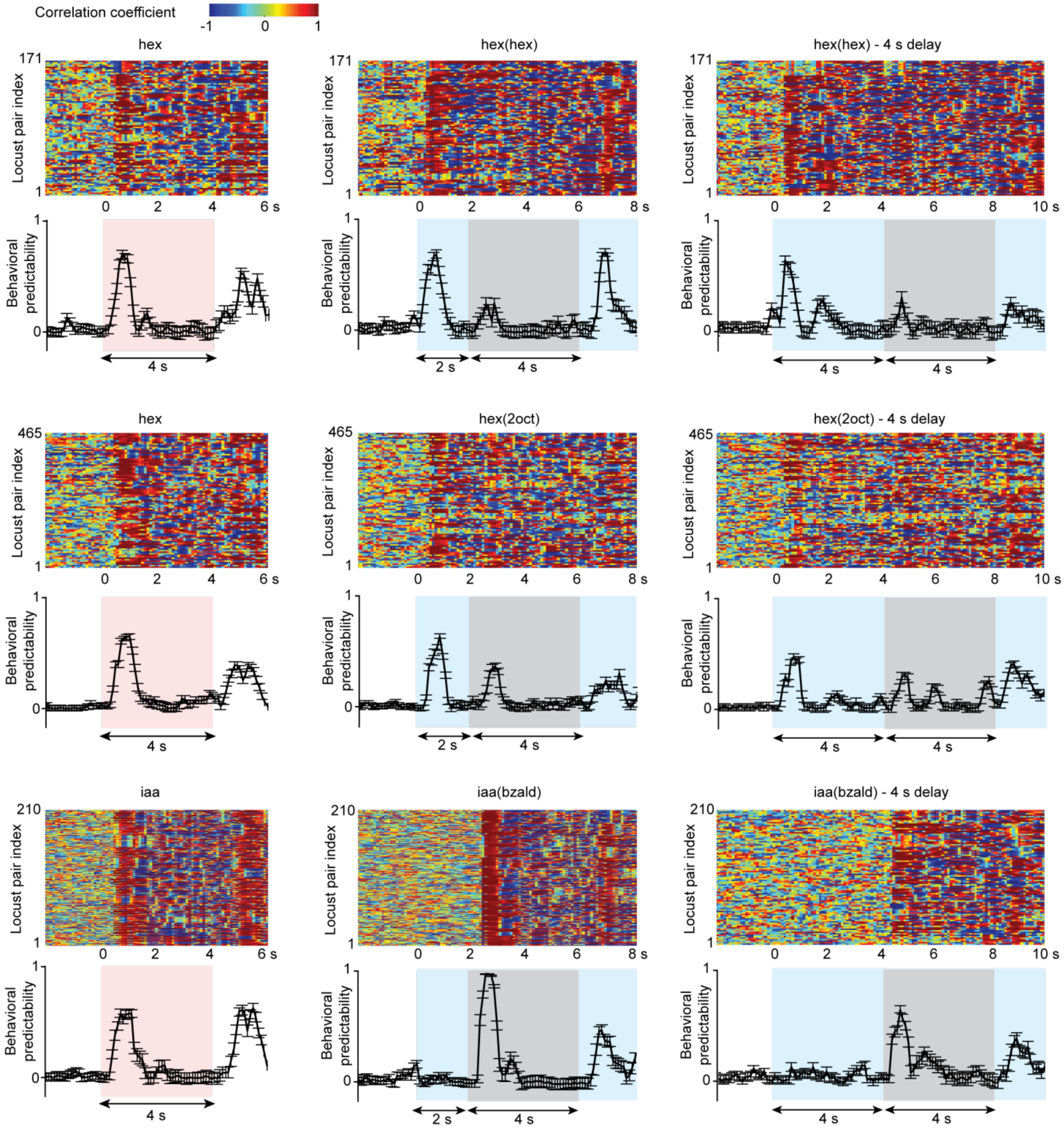

Supplementary Figure 9. Pairwise correlations between locust PORs. Top panel, pairwise correlations between all possible combinations of locust PORs are shown (see Methods). Results for all three stimuli used during test trials are shown here: conditioned stimulus presented solitarily (leftmost column), untrained odor – 2s delay – trained odor (middle column), and untrained odor – 4s delay – trained odor (rightmost column). Bottom panel, mean ± s.e.m. of pairwise correlation (computed from results shown in the top panel) is shown for each condition.

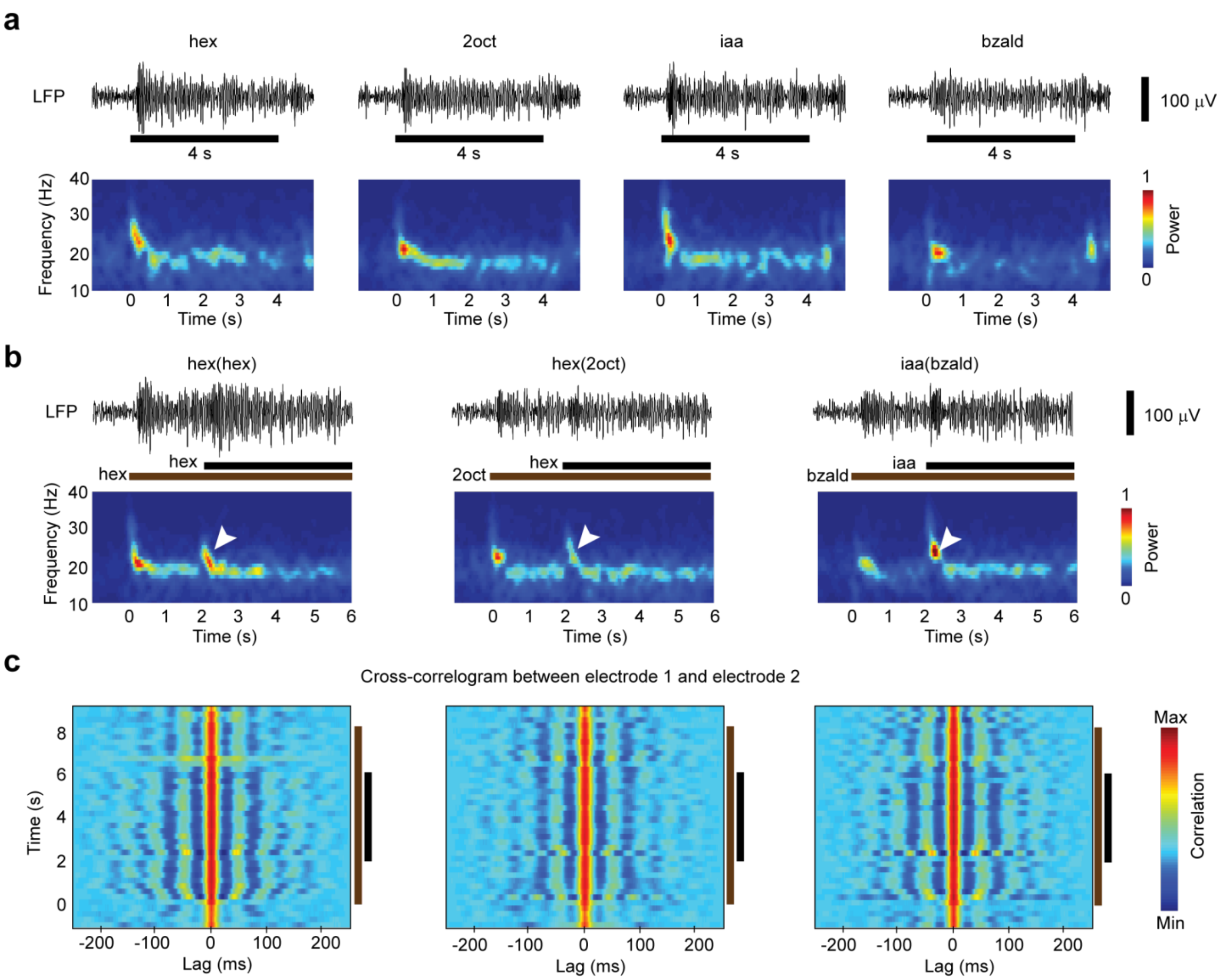

Supplementary Figure 10. Odor-evoked oscillatory synchronization in the antennal lobe. (a) Top panel, representative field potential traces (filtered between 10 – 50 Hz) obtained by placing electrodes deep in the mushroom body are shown for four different odors. Bottom panel, spectrogram showing temporal evolution of power in different oscillatory frequencies before, during and after stimulus presentations. Note, spectrograms obtained for each trial was normalized and averaged across trials and experiments (see Methods). **(b)** Similar plots as shown in **panel a,** but for overlapping odor presentations. The brown and black bars identify the epochs when the two odors were presented in an overlapping sequence. Arrows indicate increases in oscillatory power at higher frequencies following the onset of the second odor in the sequence. Note that the oscillatory synchronization persists during the entire duration of the two odor sequences. **(c)** Average cross-correlogram (250 ms non-overlapping window, 10 trials) between LFP signals simultaneously acquired from two electrodes during different two-odor sequences. The oscillatory LFP signals obtained from different electrodes were highly coherent.

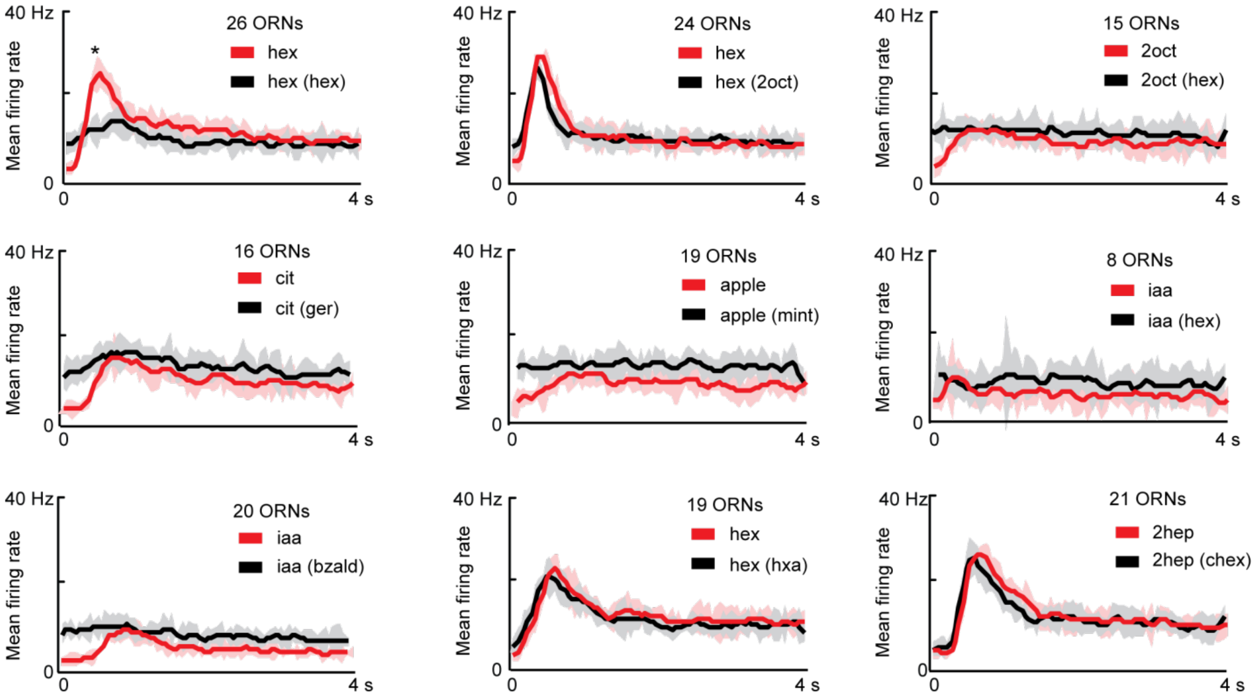

Supplementary Figure 11. Olfactory sensory neuron responses to solitary and overlapping stimulus presentations. Sensory neuron responses averaged across neurons and trials are shown for solitary (red) and overlapping (black) presentations of odor pulses. A significant reduction in peak firing rate was observed only for the second pulse of hexanol (paired t-tests; * $P = 7.5\times10^{-5}$. $n = 5$ trials).

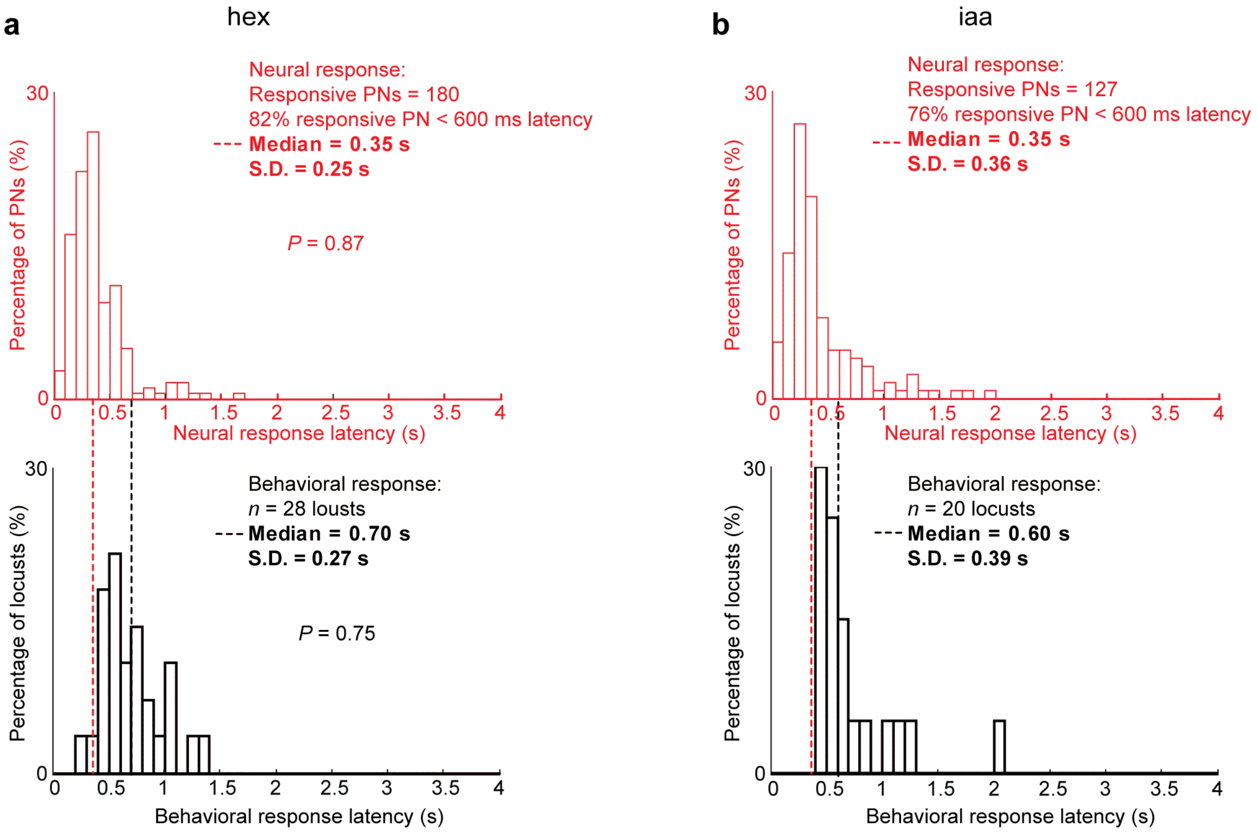

Supplementary Figure 12. Comparison of neural and behavioral response latencies. (a) *(Top panel)* PN response latency distribution is shown for hexanol (hex). Note that all neurons recorded for solitary odor introductions were used for this analysis. As can be noted, a majority of projection neurons respond within 600 ms of stimulus onset. The median latency is 350 ms. (*Bottom panel*) Similar plot but now showing behavioral response latency distribution for hexanol. As can be noted, the median behavioral response latency is 700 ms. **(b)** Similar plots as in **panel a,** but revealing neural and behavioral response latency distributions for another odorant: isoamyl acetate (iaa). Note that the neural and behavioral latency distributions of hexanol and isoamyl acetate are not statistically different (Wilcoxon rank-sum test; $P_{\text{hex vs iaa (neural)}} = 0.87$, $P_{\text{hex vs. iaa (behavior)}} = 0.75$).

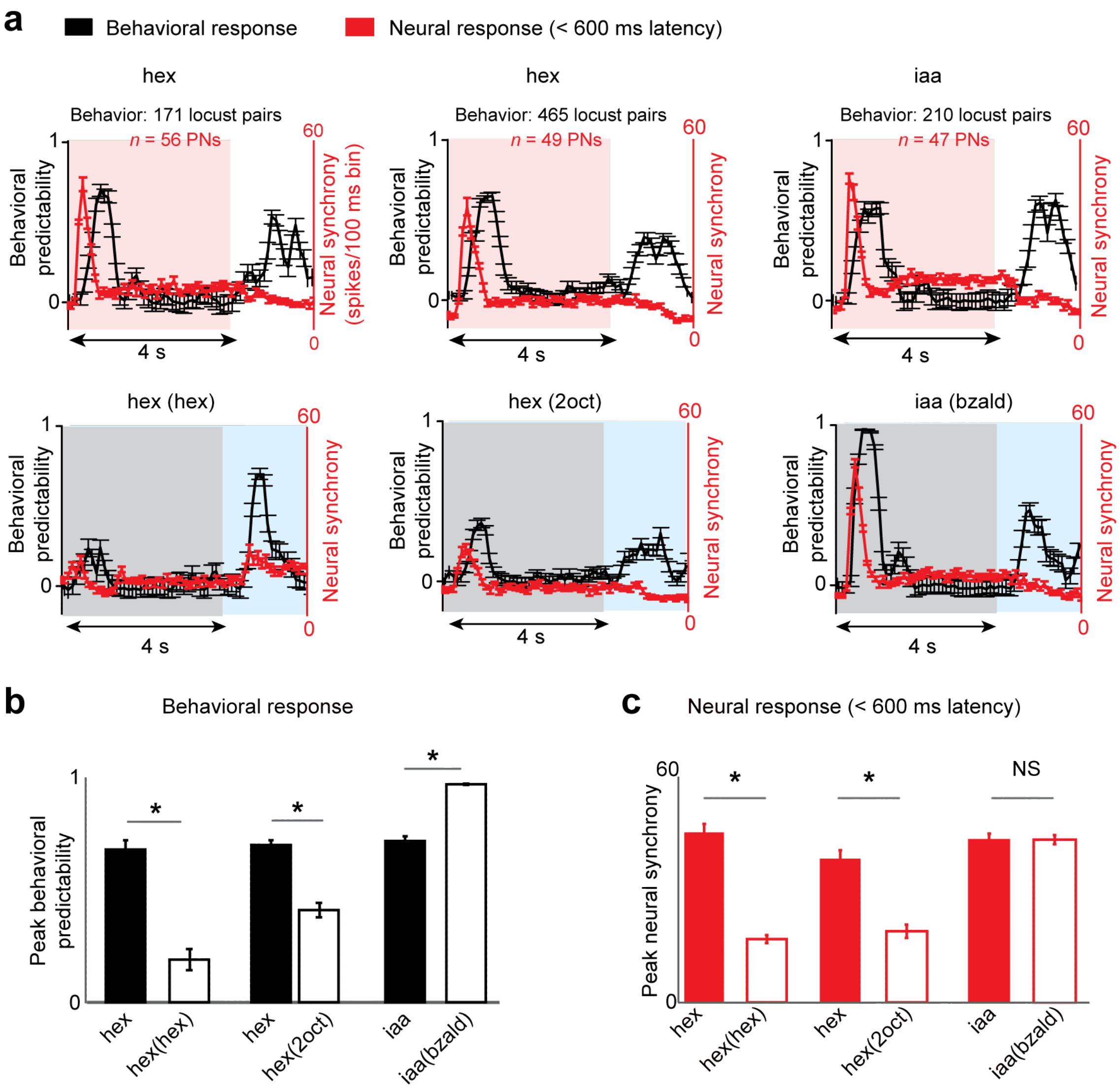

Supplementary Figure 13. Relevance of 'low latency' neurons to the behavioral response onset. Same analysis as in Fig. 5 but using only those projection neurons with response latency less than 600 ms.

Supplementary Table 1. Statistics of projection neuron response latencies for different odors.

Odor name	Median response latency (ms)	Percentage of responsive PNs with response latency < 600 ms
hexanol	350	82.8
isoamyl acetate	350	76.4
2-octanol	300	82.9
apple	400	78.4
citral	550	60.0
2-heptanone	300	91.1
mint	350	73.3
geraniol	400	69.1
benzaldehyde	300	76.7
hexanal	350	66.7
cyclohexanone	350	78.1

Supplementary Table 1. Percentage of projection neurons with response latency less than 600 ms are shown for different odorants used in the study. Note that only responses to solitary introduction of these odorants were considered for this analysis.
